# Efficient homology-directed gene editing by CRISPR/Cas9 in human stem and primary cells using tube electroporation

**DOI:** 10.1038/s41598-018-30227-w

**Published:** 2018-08-03

**Authors:** Xiaoyun Xu, Dongbing Gao, Ping Wang, Jian Chen, Jinxue Ruan, Jie Xu, Xiaofeng Xia

**Affiliations:** 1 Chao Center for BRAIN, Department of Systems Medicine and Bioengineering, Houston Methodist Research Institute, Houston, Texas USA; 2 Department of Pathology and Laboratory Medicine, University of Pennsylvania Perelman School of Medicine, Philadelphia, Pennsylvania USA; 3 Celetrix Biotechnologies, Manassas, Virginia USA; 4 Center for Advanced Models and Translational Sciences and Therapeutics, University of Michigan Medical School, Ann Arbor, MI 48109-2800 USA; 5 Weill Cornell Medical College, Cornell University, New York, New York, USA

## Abstract

CRISPR/Cas9 efficiently generates gene knock-out via nonhomologous end joining (NHEJ), but the efficiency of precise homology-directed repair (HDR) is substantially lower, especially in the hard-to-transfect human stem cells and primary cells. Herein we report a tube electroporation method that can effectively transfect human stem cells and primary cells with minimal cytotoxicity. When applied to genome editing using CRISPR/Cas9 along with single stranded DNA oligonucleotide (ssODN) template in human induced pluripotent stem cells (iPSCs), up to 42.1% HDR rate was achieved, drastically higher than many reported before. We demonstrated that the high HDR efficiency can be utilized to increase the gene ablation rate in cells relevant to clinical applications, by knocking-out β2-microglobulin (B2M) in primary human mesenchymal stem cells (MSCs, 37.3% to 80.2%), and programmed death-1 (PD-1) in primary human T cells (42.6% to 58.6%). Given the generality and efficiency, we expect that the method will have immediate impacts in cell research as well as immuno- and transplantation therapies.

## Introduction

Programmable nuclease technologies have shown great power in disease modeling and gene therapy^1^. Among these technologies the clustered regularly interspaced short palindromic repeats (CRISPR)/CRISPR-associated protein 9 (Cas9) has now become the tool of choice thanks to its simplicity and versatility^2,3^. However, the efficiency of CRISPR/Cas9 remains to be improved in order to broaden applications and eventually translate to the clinic^4^. Firstly, although high levels of gene disruption can often be achieved via NHEJ in cell lines, the efficiencies in the more clinically relevant human stem cells and primary cells are usually substantially lower. For example, in human iPSCs the overall gene disruption rate using a single guide RNA (gRNA) is typically only between 1–25% without subsequent selection^5–7^. In primary human T cells the efficiencies have been reported to be 4- to 10-fold lower than HEK293T cells for the various gRNAs and transfection methods tested^8,9^. Secondly and more importantly, there is necessity to improve the efficiency of precise gene modification via HDR, which generally occurs at significantly lower rate than NHEJ and account for no more than one-third (usually much lower) of the total editing events^10,11^. At such efficiencies, subsequent selection or subcloning is required to isolate the edited cells for further studies^12^, which it is often unsuitable for clinical applications. Techniques for increasing the CRISPR/Cas9 gene editing efficiency in clinically relevant human stem cells and primary cells are highly desirable.

Successful delivery of sufficient amount of CRISPR/Cas9 elements into the target cells by transfection is a prerequisite for efficient gene editing. Transfection methods can be broadly classified into viral, chemical and physical. Among them electroporation is the most widely used physical method. First introduced in 1982^13,14^, electroporation is easy to perform and is generally applicable to a wide range of cell types. Not requiring additional viral or cytotoxic chemical components, electroporation also is uniquely advantageous in clinical applications. However, with the high electric field strength and ensued electrochemical reactions, electroporation often leads to high post-transfection mortality. Moreover, despite the optimization of electrical parameters and solution recipes^15,16^, its efficiency on many cell types especially primary human cells is still not sufficiently high, posing a major obstacle for its clinical applications. Here we report a tube electroporation method capable of delivering nucleic acids and proteins into a diverse array of cells, including the hard-to-transfect human stem and primary cells with a very high efficiency and a very low cytotoxicity. We also demonstrate successful genome editing using CRISPR/Cas9 elements delivered by the tube device. Surprisingly, our data indicated that upon efficient delivery of the CRISPR/Cas9 elements, HDR can take place at very high rate when it is done through a single ssODN template harboring a single base pair mutation in the protospacer adjacent motif (PAM) sequence. The tube electroporation technique and the high HDR rate phenomenon may find broad clinically significant applications.

## Results

### Electroporation Tube design

**Figure 1:**
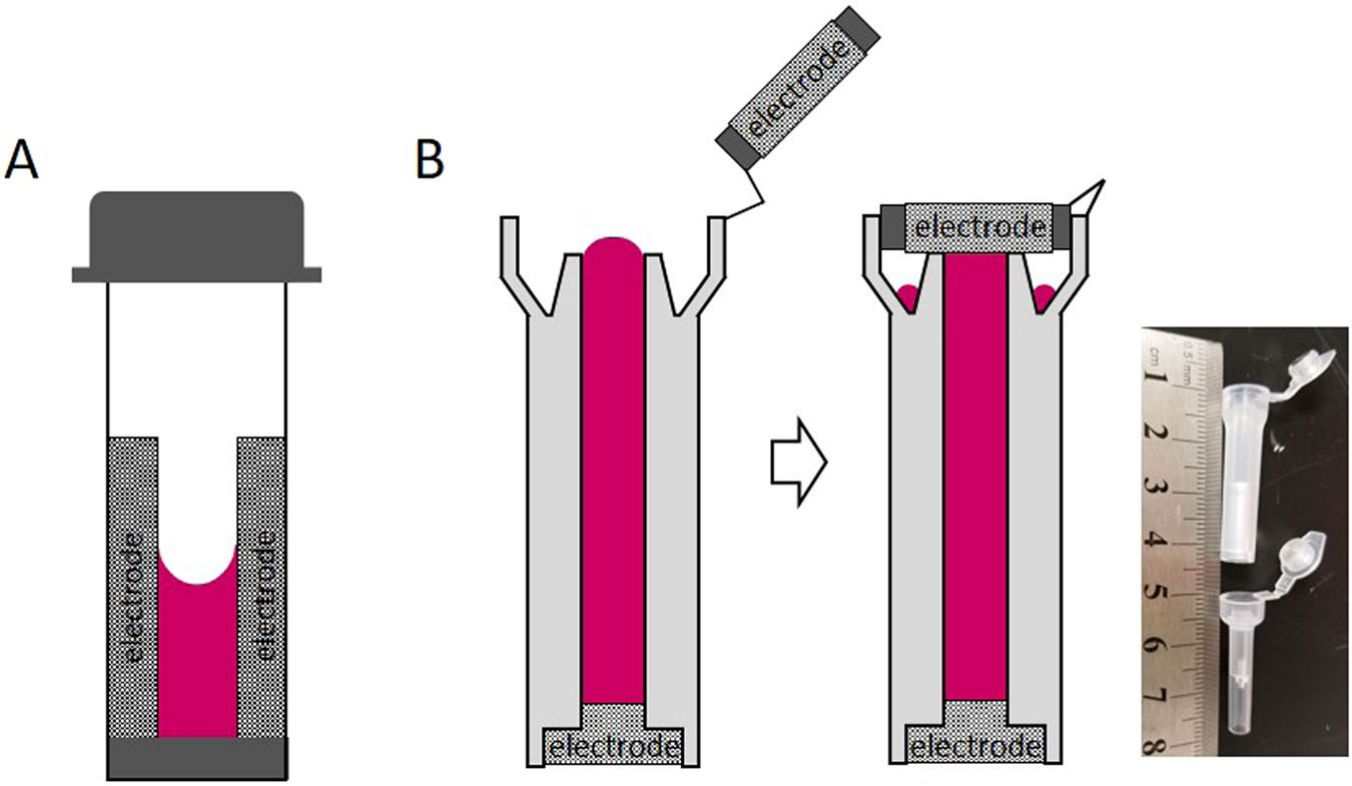
Design of the electroporation tube. (**A**) Illustration of a conventional cuvette is highly uneven in two regions. (**B**) Illustration of the electroporation tube. The tube design uses two small surface electrodes at the top and bottom. Upon closure excess liquid is pushed into the surrounding groove, creating perfectly flat top surface. This design eliminates the curved surface and minimizes the air bubble generating area, to achieve a highly homogeneous electric field within the tube. A picture of the tubes (upper: 120 μl; bottom: 20 μl) is shown on the right. The numbers shown on the ruler is by centimeter (cm).

Most current electroporation devices use cuvettes to deliver the electrical pulse to the cells (Fig. 1A), which is associated with surface warping. We reasoned that such surface warping may cause uneven voltages across the buffer. To address this concern, we designed a novel pressured electroporation tube device (Fig. 1B), with two small electrodes placed in the tube bottom and in the top cap. The tube is filled until a convex meniscus occurs. Upon closing the cap, the excess liquid is driven into the surrounding groove to generate a perfectly flat surface, therefore eliminating the surface warping effect.

### DNA transfection with tube electroporation

**Figure 2:**
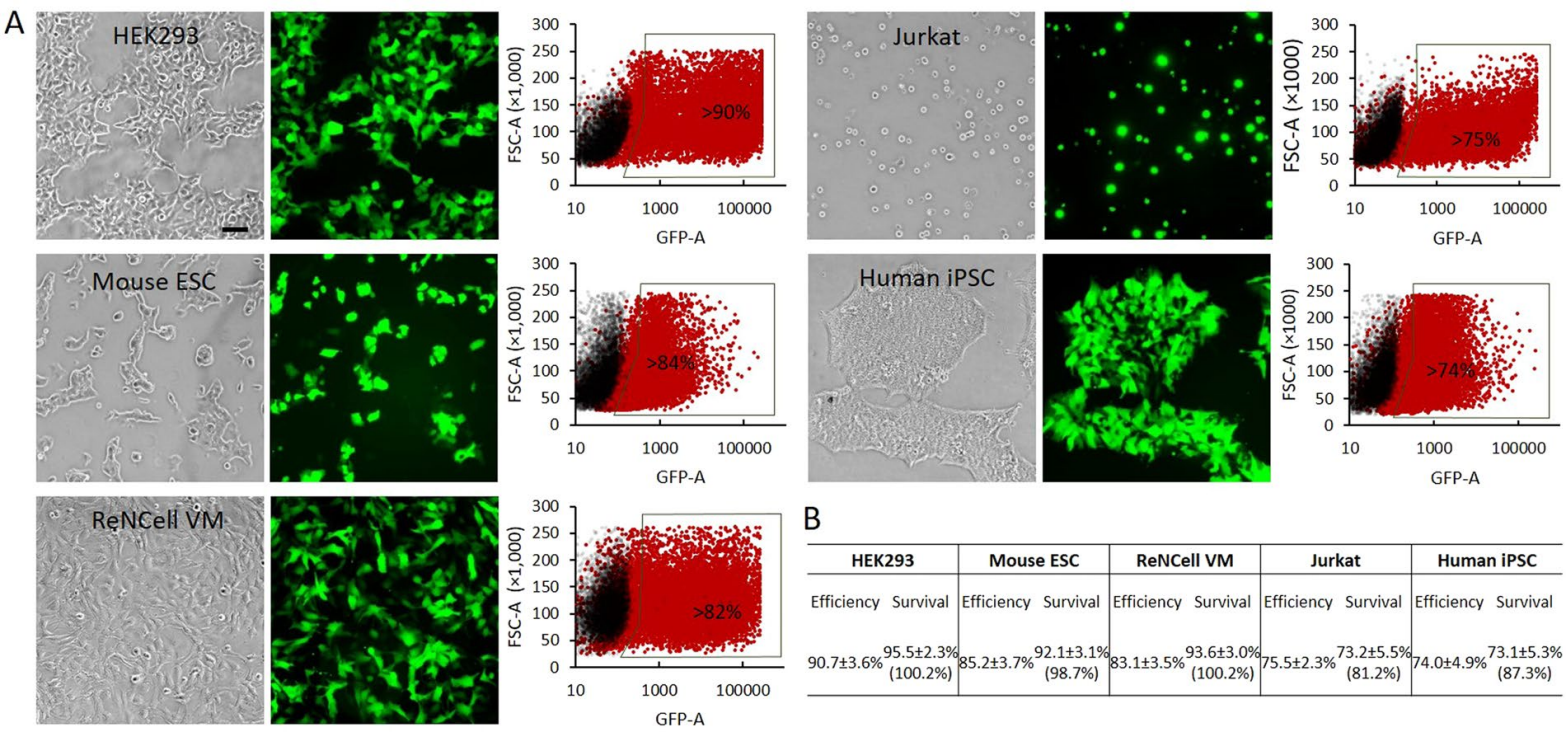
DNA transfection using tube electroporation. (**A**) Different cell types were transfected with pCMV-GFP plasmid and the transfection efficiencies were measured by fluorescence microscopy and flow cytometry after 48 hours. From left to right are the cell bright field images, fluorescence images and flow cytometry dot plots in which the transfected cells are shown in red and mock transfected control cells were shown in black. The black and red dots were from different data sets that have been juxtaposed to show the gating. Scale bar, 20 µm. (**B**) Quantification of the transfection efficiency and cell survival rates. Results are shown as mean ± S.E.M. from at least three independent experiments. The efficiencies were calculated from the GFP flow cytometry analysis. The actual cell survival rates were listed on the top. The relative survival rates compared to the untransfected controls were given in parenthesis at the bottom. The survival rates for the untransfected control cells during regular passage were 95.3 ± 2.1%, 93.3 ± 0.7%, 93.4 ± 2.9%, 90.1 ± 3.6%, and 83.7 ± 4.4% for HEK293, mouse ESC, ReNCell VM, Jurkat and human iPSC cells.

We first tested the tube device for DNA transfection, by electroporating 30 nM pCMV-GFP vector into mammalian cells and measuring the rate of GFP expressing cells and cell viability. The results showed that high efficiencies were not only achieved for cell types that are prone to electroporation such as HEK293 cells (90.7%) and mouse embryonic stem cells (ESC, 85.2%), but also for cell types that are generally considered hard-to-transfect, such as human neural stem cells (ReNCell VM, 83.1%), Jurkat cells (75.5%) and human iPSC (74.0%) (Fig. 2A,B, a complete list of cell type tested is provided in Supplemental Table 1). High mortality is a major disadvantage of conventional cuvette-based electroporation compared to other transfection methods such as using lipids or cationic polymers^17^. We analyzed in Fig. 1A and reasoned that the high voltage regions generated by surface warping and gas bubbles is a major contributor to cell mortality, which should be largely avoided in the pressured tube electroporation method. Indeed our result (Fig. 2B) confirmed that for many cell types such as HEK293, mouse ESC and ReNCell VM cells, the mortality of the transfected cells were negligible. For other more vulnerable cell types such as Jurkat cells and human iPSC, very low cytotoxicity was detected with a survival rate more than 80% compared to the untransfected control cells (Jurkat transfected VS control = 73.2% VS 81.2%; human iPSC transfected VS control = 73.1% VS 87.3%). Both the efficiency and the survival rate clearly surpass those achieved by current widely-used electroporation instruments using the manufacturer suggested or published conditions (Supplemental Table 2)^18^. Besides, we also detected a higher protein expression level after DNA transfection with tube electroporation compared to other methods (Supplemental Fig. 1).

### Protein and RNA transfection with tube electroporation

**Figure 3:**
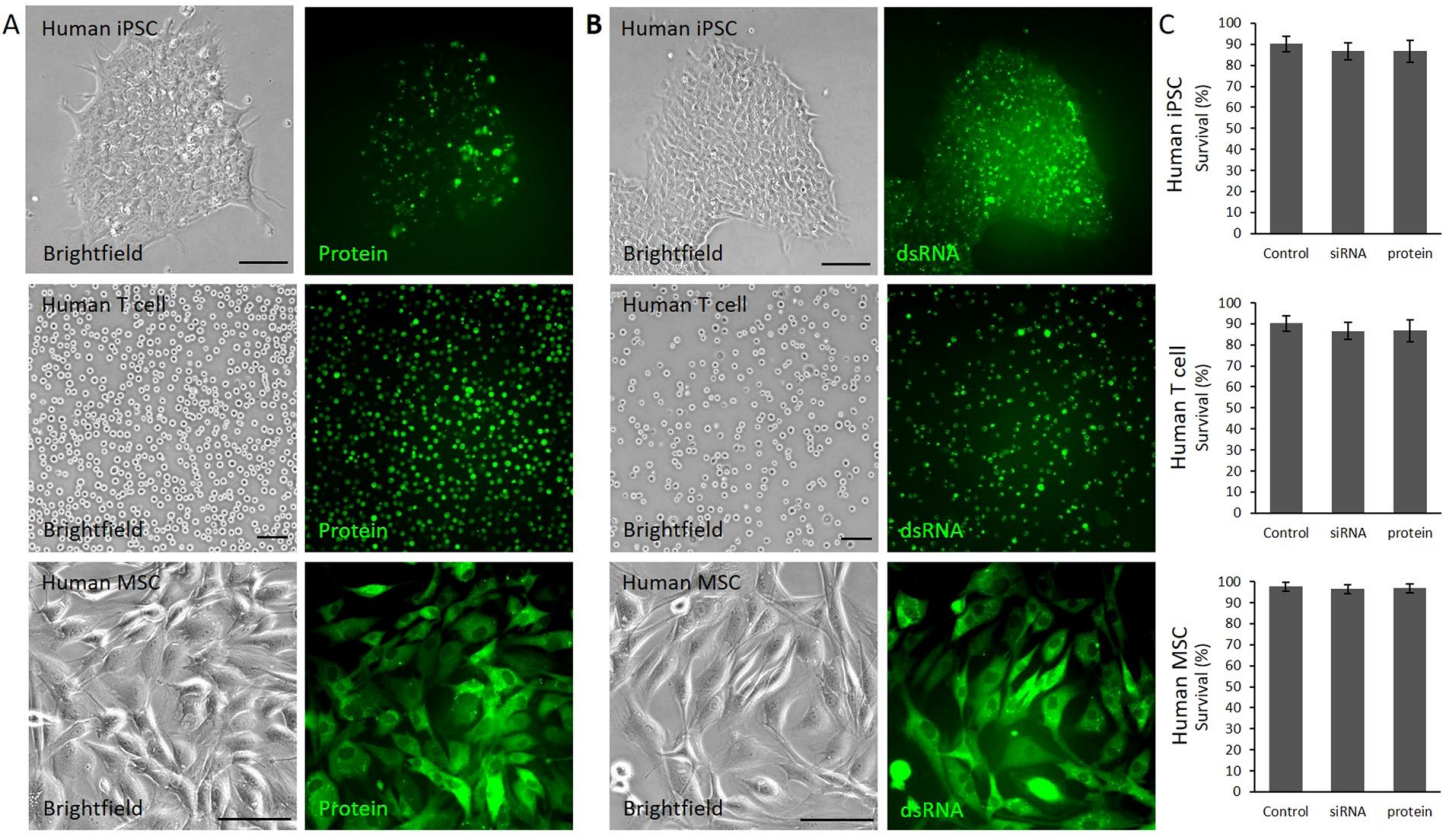
Protein and dsRNA transfection using tube electroporation. (**A**) Transfection of Alexa Fluor 488 conjugated protein (IgG antibody) into different human stem and primary cells. The brightfield images were shown in the left and fluorescence images shown in the right. (**B**) Transfection of FAM conjugated 21-mer dsRNA into different human stem and primary cells. The brightfield images were shown in the left and fluorescence images shown in the right. (**C**) Comparison of the protein and dsRNA transfected cell survival rates with untransfected controls.

We next tested the tube electroporation method for delivering protein and small double strand RNA (dsRNA) into mammalian cells. Three cell types of high clinical and research interest - human iPSC, human primary T cells and human primary mesenchymal stem cells (MSC) were selected for the test. Cells were electroporated with 0.5 μM Alexa fluor 488 conjugated goat anti rabbit IgG protein or 0.5 μM FAM conjugated synthetic 21-mer dsRNA. The result showed that both protein and dsRNA were successfully delivered into nearly every cell (Fig. 3A,B, Supplementary Figure 2). Possibly because of the smaller molecular sizes, protein and small dsRNA required lower voltage than DNA plasmid to be efficiently delivered into the cells (Supplemental Table 1), consequently the posttranfection mortality is very low or negligible for all the three cell types tested (Fig. 3C).

### CRISPR/Cas9 genome editing using tube electroporation

**Figure 4:**
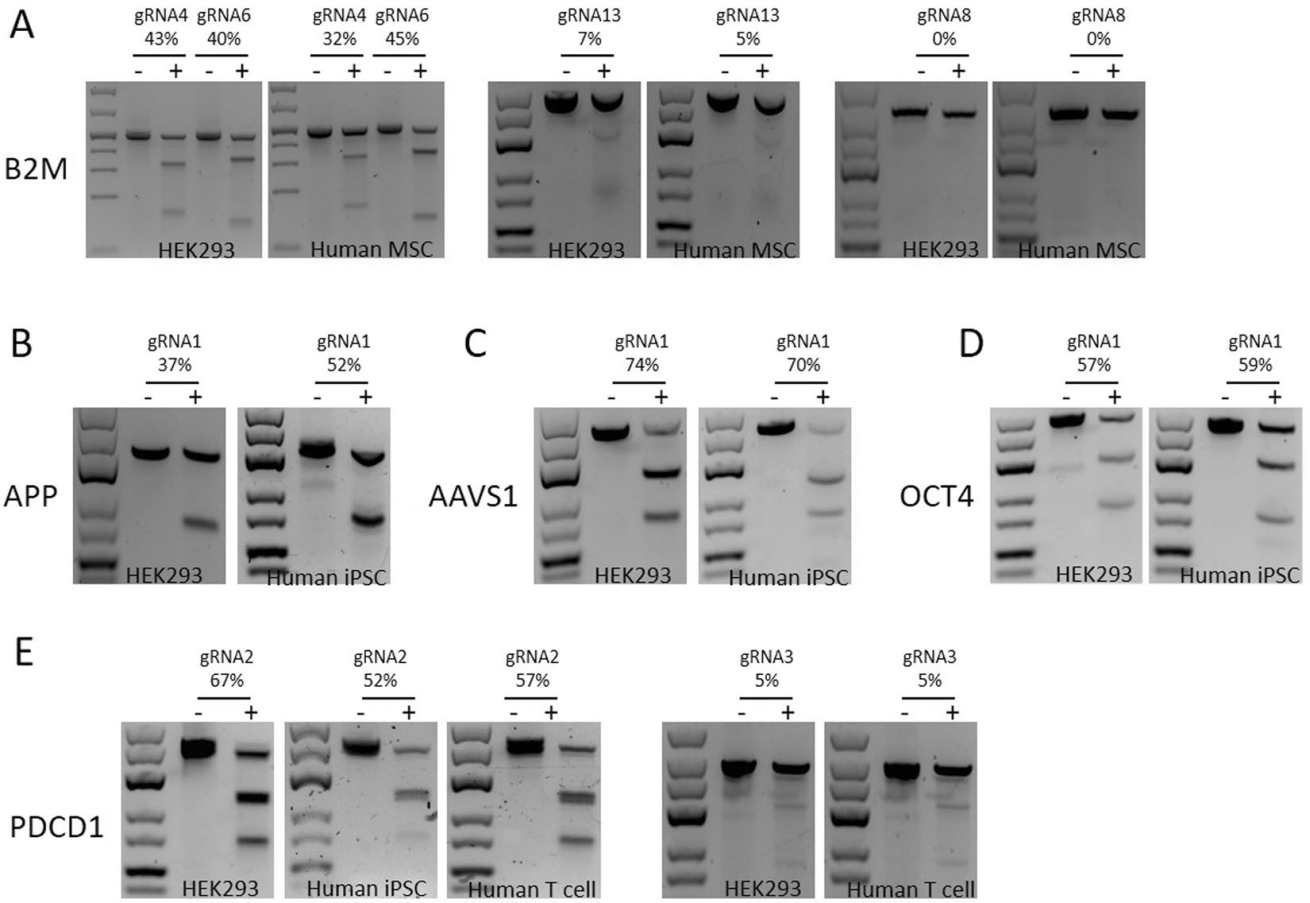
Genome editing by Cas9/gRNA RNP tube electroporation. Cells were transfected with Cas9/gRNA (+) or Cas9 only as control (−). DNAs were extracted and measured by T7EN1 assay. The average targeting efficiency of three experiments were shown above the representative gel picture. (**A**) Four gRNAs were tested targeting B2M gene in HEK293 and human MSCs. gRNA4 and gRNA6 were shown to be effective in both cells (efficiencies >30%), gRNA13 was weakly effective in both cells (efficiencies <10%), while no activity was detectable for gRNA8 in both cells. (**B**) Targeting APP gene in HEK293 and human iPSC. (**C**) Targeting the AAVS1 site in HEK293 and human iPSC. (**D**) Targeting OCT4 gene in HEK293 and human iPSC. (**E**) Two gRNAs were tested targeting PDCD1 gene in HEK293, human iPSC and primary human T cells. gRNA2 were shown to be effective in all three cell types (efficiencies >50%), while the activities of gRNA3 were very weak (efficiencies = 5%) in both HEK293 and human T cells. In all these experiments the efficiency of the same gRNA is very consistent in different cell types with a variance <30%.

CRISPR/Cas9 has rapidly become the method of choice for generating gene knock-out or knock-in cells. A highly efficient low cytotoxicity tool for delivering the CRISPR/Cas9 elements, including the Ca9 protein, gRNA and a DNA repair template when applicable into mammalian cells would greatly facilitate its clinical and research applications. We tested the tube electroporation method for transfecting Cas9/gRNA ribonucleoprotein (RNP), which has been shown to achieve efficient and specific genome editing in mammalian cells recently^9,19,20^. We used gRNAs that were previously reported by others (Supplemental Table 3) to target 5 different gene loci - B2M^8^, amyloid precursor protein (APP)^10^, Adeno-associated virus integration site 1 (AAVS1), octamer-binding transcription factor 4 (OCT4)^21^ and PDCD1^22^ in 4 types of cells – HEK293FT, primary human MSC, human iPSC and primary human T cells (Fig. 4). The results showed that tube electroporation allowed efficient RNP delivery leading to high levels of gene editing for all of the genes in all the cells. Consistent with literature reports, we observed the gene targeting efficiency to be highly gRNA specific, as even partially overlapping gRNAs displayed significantly different results in the same type of cells. We also observed a consistency of efficiencies of the same gRNA in different cell types, as each gRNA achieved close levels of gene targeting in different cells, ranging from 0% to 70%. Early work indicated that the activity of CRISPR/Cas9 may be remarkably lower in primary human cells, with a up to 10-fold efficiency reduction compared to HEK293T cells^8,9^. However, our current result suggests that upon efficient RNP delivery, gRNA efficiency is highly consistent (variance <30%, Fig. 4) in the different cell types that we tested. The high disparity reported previously may possibly be attributed to insufficient DNA delivery in primary human cells and/or the difference in Cas9 protein translation in different cell types. Therefore these results support that tube electroporation of RNP may be used as a more efficient delivery method for CRISPR/Cas9, especially for the hard-to-transfect human stem cells and primary cells.

### High HDR by RNP tube electroporation

**Figure 5:**
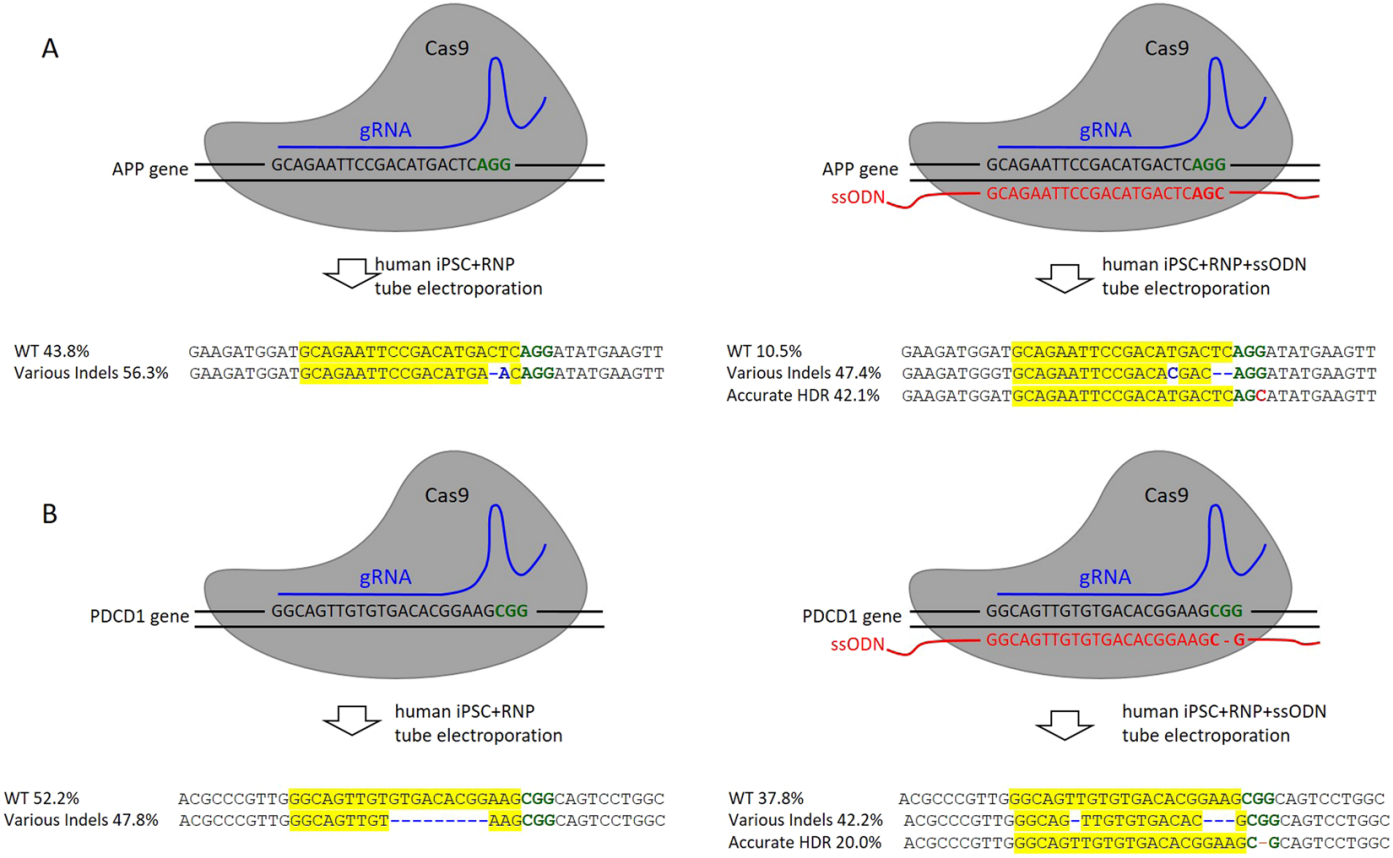
Tube electroporation achieves high accurate HDR rate in human iPSC. (**A**) Human iPSC were transfected with Cas9/gRNA RNP targeting APP gene, without (left) or with (right) an ssODN template. DNA sequencing showed that the editing efficiency was 56.3% without ssODN, and all the edited cells have Indels. The overall editing efficiency was increased to 89.5% with ssODN, including 47.4% of the total cells having various Indels, and 42.1% of the total cells having a single G to C mutation introduced by the accurate HDR with the ssODN template. (**B**) Human iPSC were transfected with Cas9/gRNA RNP targeting PDCD1 gene, without (left) or with (right) an ssODN template. DNA sequencing showed that the editing efficiency was 47.8% without ssODN, and all the edited cells have Indels. The overall editing efficiency was increased to 62.2% with ssODN, including 42.2% of the total cells having various Indels, and 20.0% of the total cells having a single G deletion introduced by the accurate HDR with the ssODN template. The sing base deletion would result in an open reading frame shift of the gene.

Fluorescently labeled protein and dsRNA indicated that the molecules can be delivered at nearly 100% efficiency using tube electroporation (Fig. 3). However, a significant amount of cells remained unedited even with the best gRNA as shown by the T7EN1 assay (Fig. 4). This was confirmed by direct Sanger sequencing of the targeted APP site in human iPSC (56.2% Indel, 9/16 clones) (Fig. 5A, Supplemental Table 4). The incomplete editing may be due to several reasons: RNP is more difficult to transfect (Cas9/gRNA RNP is about 180kd and larger than the 150kd IgG protein, or the 14kd 21-mer dsRNA) so that it was less efficiently delivered into many cells; or the RNP was inactive in these cells; or the cells were edited but then repaired by the active DNA repair machinery. To test these possibilities, we co-transfected an ssODN repair template, together with Cas9/gRNA RNP targeting APP in human iPSC. The 100nt ssODN carries a single base mutation in the PAM sequence (AGG to AGC) (Supplemental Table 3)^10^. Upon HDR PAM should be eliminated to prevent further targeting. Sanger sequencing was carried out using the PCR products of the targeted regions amplified from the genomic DNA of the bulk transfected cells. The PCR products were cloned into a pJET vector and the plasmid DNA purified from individual *E.coli* clones were used for sequencing. The sequencing results surprisingly displayed a much higher mutation rate (89.5%, Fig. 5A) than the cells transfected without ssODN (56.2%). Noteworthy is that the majority of the mutated DNAs (42.1% of the total) had the same single base pair PAM mutation but were free of any additional indel, therefore were the result of accurate HDR^10^ using the ssODN template (Fig. 5A, Supplemental Table 4). This result suggests that firstly, Cas9/gRNA RNP can be successfully delivered into nearly all (at least 89.5%) of the human iPSCs by the tube electroporation; secondly, the transfected RNP is active in human iPSC and cleaves DNA in at least 89.5% of the cells; thirdly, active DNA repair exists in human iPSC and more than 1/3 ((89.5–56.2%)/89.5% = 37.2%) of the double strand breaks (DSBs) may be accurately repaired later and appear to be unedited. NHEJ is the predominant DNA repair mechanism after DSB by CRISPR/Cas9. Because CRISPR/Cas9 typically generates high levels of gene disruption, NHEJ is mostly recognized as error-prone but this view has also been challenged^23,24^. Our current data may support the intrinsic precision of NHEJ repair at least in human iPSC at the targeted loci, suggesting that most Indels may be accurately repaired and mutations are only introduced after repetitive DSB/repair processes. Our data also demonstrated a surprisingly high accurate HDR rate in human iPSC (42.1% of the total unselected transfected cells). Efficient RNP/ssODN delivery by the tube electroporation is apparently an essential contributor for the high HDR rate. Previously the same gRNA and ssODN was transfected into human iPSC by other electroporation method and only 2.1–6.7% HDR rate was observed in the Cas9-GFP sorted cells^10^. The tube electroporation method improved the efficiency by an order of magnitude.

The high HDR rate by tube electroporation may greatly facilitate the generation of single-base genome edited (i.e. point mutation) cells in which the isolation of rare clones is often hindered by the unavailability of antibiotic selection^12^. Among the human DNA point mutations that cause pathological phenotypes, a vast amount either lead to the ablation of an existing PAM or creation of a new PAM (Supplemental Table 5) and can thus be studied by the current method. However, the application is limited to mutations in PAM sequences because it is dependent on the preventing of further targeting by HDR. A more universal application of the high HDR rate at PAM sites may be the introduction of single base pair insertion or deletion to create a frame shift, thereby knock-out the gene expression^20^. As our result indicated that accurate NHEJ repair may significantly reduce the number of cells with Indels, and virtually any gene exon would have PAM sequence (NGG or CCN), this method may be used universally to increase the gene knockout rate by combing the HDR mediated frame shift and NHEJ mediate Indel. To test this possibility we chose the gRNA targeting the PD-1 encoding human PDCD1 gene (Fig. 5B) that is now actively being studied in immune checkpoint blockade therapy^25^. Human iPSC were transfected using tube electroporation with Cas9/gRNA RNP only, or together with an ssODN carrying a single base deletion in the PAM site (CGG to CG, Fig. 5B). Sequencing of the genomic DNA extracted from the transfected cells showed that 47.8% of the DNAs are mutated (11 out of 23 clones) with RNP alone. Inclusion of the ssODN significantly increased mutation rate to 62.2% (28 out of 45 clones). Again, a marked percentage (20.0%, 9 out of 45 clones) of the mutations resulted from accurate HDR, which introduced a single base deletion in PAM (Fig. 5B, Supplemental Table 6) and would cause a frameshift. Together, these results confirmed that upon efficient RNP delivery by tube electroporation, HDR occurs in human iPSC at surprisingly high rate, and can be explored to create mutation knock-in cell lines or increase the rate of gene disruption.

### Ablation of clinically relevant genes in primary human cells

**Figure 6:**
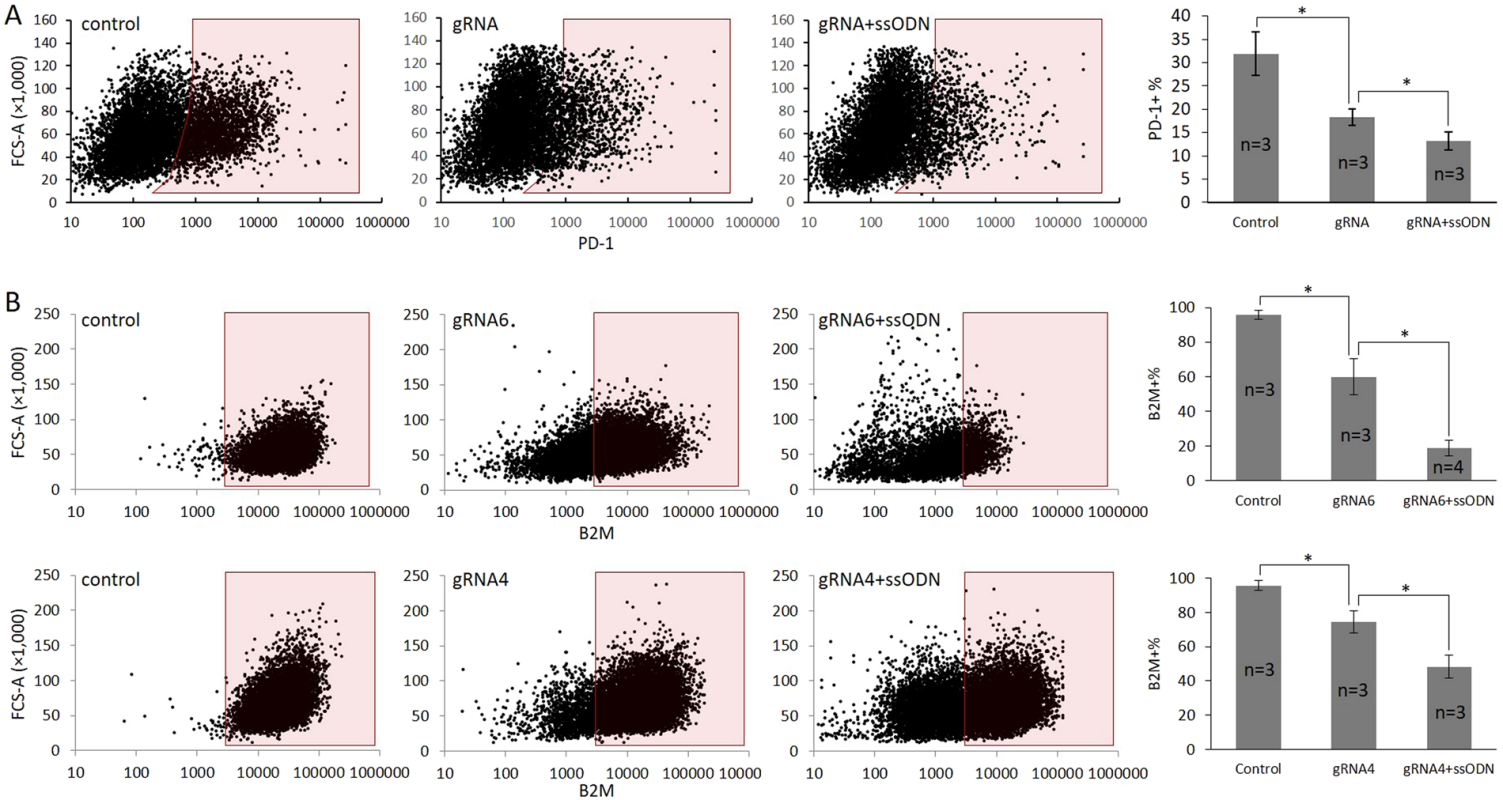
Enhancing the gene knock-out rate in primary human cells by ssODN HDR. Cell surface protein expression was analyzed by flow cytometry (left) and quantified (right). (**A**) Knocking-out PD-1 in primary human T cells. Tube electroporation of the Cas9/gRNA RNP reduced the surface expression of PD-1 from 31.9% to 18.3%. Inclusion of a frameshift introducing ssODN (a single G deletion in PAM) further reduced it to 13.2%. (**B**) Two gRNAs (gRNA6 and gRNA4) were selected to knock-out B2M gene in human MSCs. For gRNA6 (upper), tube electroporation of the Cas9/gRNA RNP reduced the surface expression of B2M from 95.6% to 59.9%. Inclusion of a frameshift introducing ssODN (a single C insertion in PAM) further reduced it to 18.9%. For gRNA4 (lower), tube electroporation of the Cas9/gRNA RNP reduced the surface expression of B2M from 95.9% to 74.4%. Inclusion of the ssODN further reduced it to 48.3%. The numbers of repeating experiments were indicated in each bar graph.

High gene knock-out rate is often desired in therapeutic applications, in which pre-selection is usually unavailable and high editing rate is critical for better clinical outcome^4^. For example, autologous transplantation of PD-1 knockout T cells are now being studied in several clinical trials (e.g. NCT02793856 for metastatic non-small cell lung cancer and NCT02867345 for castration resistant prostate cancer)^26^, as an alternative checkpoint blockade strategy to the costly antibody treatment. We have shown that PDCD1 gene can be efficiently targeted in human iPSC (Fig. 5), but the effect on PD-1 expression could not be tested as it is not expressed in the undifferentiated human iPSC. To demonstrate the effect on protein expression and test the potential of the tube electroporation method in immunotherapy, activated CD3+ T cells were electroporated with Cas9 RNP, with or without a ssODN carrying the single nucleotide deletion mutation (Fig. 6A). Flow cytometry confirmed that this strategy is indeed effective in reducing PD-1 expression in CD3+ T cells. The percentage of PD-1 expressing cells was reduced from 31.9% to 18.3% (42.6% reduction) without using ssODN. Use of PAM mutated ssOND, as expected, further reduced percentage of the PD-1 expressing cells to 13.2% (58.6% reduction).

Human MSC is numerically is the most favored cell type presently under clinical trial^27^. Genetic modification of the cells would further broaden its therapeutic potential and enhance its safety. Genome editing of primary human MSCs has been achieved using adenoviral Cas9^28^, but non-viral method has not been reported. We tested the tube electroporation for knocking-out B2M, a well-established MHC class I molecule association protein, in human MSCs. Several gRNAs were tested and two of them (gRNA4 and gRNA6) were shown to be effective by T7EN1 assay (Fig. 4A). The RNP was electroporated into the cells with or without an ssODN template, which introduces a single base (C) insertion in the PAM sites and creates a frameshift mutation to disrupt gene function. Flow cytometry confirmed the enhancement of gene disruption rate by the ssODN. For gRNA4, the cell-surface B2M expressing cells were reduced from 95.9% to 74.4% (22.4% reduction) by Cas9/gRNA alone, and further reduced to 48.3% (49.6% reduction) by the inclusion of ssODN. gRNA6 resulted in a more significant gene disruption and reduced B2M expressing cells from 95.6% to 59.9% (37.3% reduction) by Cas9/gRNA alone, and further reduced to 18.9% (80.2% reduction) by the additional ssODN (Fig. 6B). Together these results strongly support that tube electroporation can effectively deliver RNP/ssODN, to achieve high HDR universally in different cell types, and can be utilized as a general method to enhance gene knock-out rate in different types of cells.

## Discussion

We show here that a novel tube electroporation method can deliver CRISPR/Cas9 RNP components without or without ssODNs at remarkably high efficiency into various human stem cells and primary cells that are hard-to-transfect. It also leads to satisfactory knock-in results when donor DNA is of large size (e.g. a reporter gene) at a level comparable to that achieved by the popular Nucleofector machine (Supplementary Figure 3).

Electroporation transfection method was developed 35 years ago^13,14^. In the past many improvements have been made, for example, the nucleofector technique^15,16^ that provided the optimization of electrical parameters and solution recipes. Notably, most current electroporation devices use cuvettes to deliver the electrical pulse to the cells. Despite of its long history and wide usage, the cuvette suffers from physical effects that have been overlooked. A primary concern is the surface warping effect. Due to surface adhesion, a concave meniscus naturally occurs in the cuvette (Supplementary Figure 4, region 1). As the cuvette is often only filled partially at the bottom and the space between the electrodes is narrow (typically 1- to 4-millimeter), it is conceivable that a substantial portion of the sample is close to the concave meniscus with an uneven electric field distribution. The voltage distribution in the liquid can be simulated by a simplified equivalent circuit (Supplementary Figure 4, region 1). The whole liquid can be subdivided into identical resistors that are connected into multiple series in parallel with the same end-to-end total voltage. Since in a series circuit voltage is distributed in proportion to resistance, each of these resistors would have the same voltage if the liquid surface is flat. However in the concave meniscus two resistors are first connected in parallel (Supplementary Figure 4, region 1 blue resistor) with the resistance cut in half and they are then connected in series to the center resistor (Supplementary Figure 4, region 1 red resistor). Therefore the side of the concave meniscus would assume a lower voltage, which is insufficient for effective transfection; while the center region beneath the concave meniscus is allocated an overly high voltage that would damage or kill the cells. Another notable adverse effect in a cuvette design is the bubble effect (Supplementary Figure 4, region 2). Electroporation inevitably generates air bubbles on the electrode surface as a result of electrochemical reactions. Because air is insulating, lower voltage regions are created on both sides of each bubble, while the electrons are forced through the spaces between bubbles creating very high voltage regions, again either insufficient for transfection or causing high cytotoxicity.

In the tube electroporator design, the surface warping effect is eliminated by placing the electrodes in a top-bottom manner (Fig. 1B). Because the surface area of the electrodes is also significantly reduced, this design minimizes air bubble regions as well. The small gas volume is further reduced by the sealing of the sample and the applied pressure to the sample from the electroporation machine and the tube itself. Based on these considerations, it is not surprising that the tube electroporation indeed leads to satisfactory transfection results as demonstrated in this study.

With the high transfection efficiency achieved in this study, a few new properties of CRISPR/Cas9 were discovered that may change our current notion. Firstly, although restricted to the PAM site, we determined that HDR can happen at unexpectedly high rate (a magnitude of order higher than previous report). This may significantly change the strategy for creating knock-in cell models, and as we demonstrated it can also be utilized as a strategy to increase the gene knock-out rate. Secondly, we observed the high editing efficiency consistently in different cell types, indicating that the previously reported cell type specific inefficiency may be due to the insufficient delivery of CRISPR/Cas9 components. Our method can also be combined with other recently improved CRISPR/Cas9 components, such as the chemically modified gRNA^29^, negative supercharge fused protein^19^, to further boost the genome editing efficiency.

We demonstrated the application of our method in introducing single base gene modification in human iPSC, and knocking-out clinically relevant genes in primary human cells. These applications may have direct impact in cell research as well as immuno- and transplantation therapies. Currently there are more than 300 registered human MSC clinical trials^27^, for various conditions including orthopedic injuries, graft versus host disease following bone marrow transplantation, cardiovascular diseases, autoimmune diseases, and liver diseases. In Canada and New Zealand, human MSCs have been approved for pediatric graft versus host disease^30^. Although human MSC is generally considered to have low immunogenicity, MSCs are not intrinsically immune-privileged and may trigger immune responses depending on the administration route and transplantation time, and possible account for their very brief existence in the host (typical only a few days, no more than a week or two)^31–33^. Protecting MSCs from immune detection and prolonging their persistence *in vivo* may improve clinical outcomes and prevent patient sensitization toward donor antigens. B2M encodes a non-membrane-anchored glycoprotein to associate with major histocompatibility complex (MHC) class I molecules and is required for their surface expression. Deletion of B2M is therefore a well-established strategy to ablate MHC class I surface expression and reduce the immunogenicity of allogeneic transplants^34^. Using tube electroporation we first achieved high efficiency non-viral transfection of CRISPR/Cas9 in human MSCs, and demonstrated that this strategy can be successfully applied to human MSCs to eliminate the surface expression of B2M in the majority of the cells without requiring any selection, thus may produce safer and longer lasting transplantation sources. Immune checkpoint blockade by PD-1 antibody has shown remarkable antitumor responses in patients with advanced melanoma, lung cancer as well as other cancer types^35^. The ability to knock-out PD-1 expression in primary human T cells opens a new revenue of adoptive cell therapy^20^, to possibly avoid the costly continuous antibody treatment. Also of note, previous studies have demonstrated that PD-1 blockage may significantly enhance the efficacy of genetically modified T cells expressing a chimeric antigen receptor (CAR)^36^. The tube electroporation method was shown to be able to eliminate PD-1 expression efficiently through CRIPSR/Cas9 mediated NHEJ and HDR. Either used alone or in combination with CAR overexpression, the method could potentially provide a useful tool to improve the efficacy of T-cell based adoptive therapies.

## Methods

### Cell culture

All the cells used in this study were purchased from commercial sources that are listed in the below sections. An ethical approval is not required for this study.

#### Human iPSC

Human iPSCs were purchased from American Type Culture Collection (ATCC, Manassas, VA 20110, ACS-1030) or Systems Biosciences (SBI, Palo Alto, CA 94303, SC600A-WT). Cells were cultured in feeder-free condition in mTeSR1 medium (StemCell Technologies, Vancouver, Canada) supplemented with 10 ng/ml bFGF (StemCell Technologies, Vancouver, Canada), on Matrigel (Corning, Corning, NY) coated cultureware surface. For transfection, cells were dissociated with Accutase (StemCell Technologies, Vancouver, Canada) to single cells. To improve survival 10 µM ROCK inhibitor Y27632 (Sigma-Aldrich, St. Louis, MO) was added to the transfected cells and removed after the cells are attached.

#### Mouse ESC and iPSC

Mouse ESC (ES-D3) were purchased from ATCC (Manassas, VA, CRL-1934). Mouse iPS cells were purchased from Systems Biosciences (SBI, Palo Alto, CA 94303, SC201A-1). Both cells were cultured in feeder-free condition in medium containing DMEM (ThermoFisher Scientific, Waltham, MA), 15% fetal bovine serum (FBS, ThermoFisher Scientific, Waltham, MA), 100 µM non-essential amino acids, 100 µM 2-mercaptoehanol, and 1,000 U/ml LIF (Peprotech, Rocky Hill, NJ). Cell cultureware were pre-coated with 0.2% gelatin (Sigma-Aldrich, St. Louis, MO). For transfection cells were dissociated with 0.25% trypsin ((ThermoFisher Scientific, Waltham, MA) to single cells.

#### Human MSC

Human MSCs derived from adipose (ZenBio, Research Triangle Park, NC), cord blood (Cellular Engineering Technologies, Coralville, IA) or bone marrow (ThermoFisher Scientific, Waltham, MA) were purchased from the indicated sources. Cells were cultured in DMEM/F12 containing 10% FBS and 10 ng/ml bFGF. For transfection cells were dissociated with 0.25% trypsin to single cells.

#### HEK293 cells

HEK293 cell line was purchased from Sigma-Aldrich (St. Louis, MO). Cells were cultured in DMEM containing 10% FBS. For transfection cells were dissociated with 0.25% trypsin to single cells.

#### Human neural progenitor cells

Human neural progenitor cell line ReNCell VM was purchased from Millipore (Billerica, MA). Human embryonic stem cell (H9) derived neural progenitor cells were purchased from ThermoFisher. Cells were culture in knockout DMEM/F12 (ThermoFisher) with StemPro supplement (ThermoFisher), 10 ng/ml bFGF and 10 ng/ml EGF (StemCell Technologies), on laminin (ThermoFisher) coated cultureware. For transfection cells were dissociated with accutase to single cells.

#### Jurkat cells

Jurkat cells were purchase from ATCC. Cells were cultured in suspension in RPMI-1640 (ThermoFisher) containing 10% FBS medium.

#### Human primary T cells

CD3+ human primary T cells were purchased from Astarte Biologics (Bothell, WA). Cells were cultured in suspension in ImmunoCult-XF T cell expansion medium (StemCell Technologies) with 10 ng/ml recombinant human IL-2 (PeproTech). To activate the cells 100 ng/ml anti-CD3 antibody (clone OKT3, eBioscience, San Diego, CA) was added to the medium. Cells were activated and expanded for two weeks before they are collected for transfection experiments.

#### Human primary hepatocyte

Metabolism qualified primary human hepatocyte was purchased from Lonza (Walkersville, MD). Cells were grown adherently in hepatocyte plating medium (Lonza). For transfection cells were dissociated with Accutase (StemCell Technologies) to single cells.

#### Primary rat cortex neural progenitor cells

Neural progenitor cells were cultured from the E18 rat brain cortex. Tissues were dissected and dissociated with Accutase (StemCell Technologies) to single cells, and grown in suspension to form neurospheres first. The spheres were then digested with Accutase to single cells and grown adherently as monolayer on 20 μg/ml laminin (ThermoFisher) coated surface. For transfection the adherent cells were dissociated with Accutase to single cells.

### Electroporation

The electroporation machine (Catalog# CTX-1500A LE), the pressured electroporation tubes (Catalog# 20 µL: 12–0107; 120 µL: 12–0104; 200 µL: 12–0101), and the electroporation buffer (Catalog# 13–0104) were provided by Celetrix LLC, Manassas VA. Single cell suspensions were prepared as indicated in Cell Culture. Cells were resuspended in electroporation buffer to 25 × 106 cells/ml. For GFP DNA transfection 30 nM pCMV-GFP plasmid (Addgene,Cambridge, MA) was added to the cells. For Cas9 ribonucleoprotein (RNP) transfection, 0.5 μM Cas9-NLS protein (PNA Bio, Newbury Park, CA) was pre-mixed with 0.85 μM gRNA at room temperature for 10 minutes first, then the formed RNP complex was mixed with the cells and transferred to 120 µl electroporation tube. In indicated experiments 2.7 μM ssODN was also added to the mixture. The electroporation conditions were listed in Supplemental Table 1. After electroporation the cells were immediately transferred back to warm medium to continue culture.

### GFP reporter knock-in

To knock in a GFP reporter provided by plasmid DNA donor, we constructed AAVS1 and Rosa26 targeting vector respectively. The GFP coding sequence was amplified from pEGFP-N1, splice acceptor (SA) sequence was added to the upstream of the GFP coding sequence, homology arms of 500 to 600 bp were amplified from the genomic DNA purified from human iPSC. AAVS1 sgRNA targeting sequence were designed to locate in the first intron of PPP1R12C, and Rosa26 sgRNA were designed to locate in the third intron of Rosa26. To knock in the GFP reporter, we co-transfected AAVS1/Rosa26-specific sgRNA, PX330 vector, and AAVS1/Rosa26 targeting vector into human iPSC by the tube electroporation and Lonza’s Nucleofector. GFP positive cells as the indicator of knock in events were analyzed by flow cytometry four days after transfection.

### Flow cytometry

Flow cytometry was done with BD FACS Fortessa instrument and analyzed with BD VACSDiva software. The data was exported to Excel to make the plots. Cells were harvested 48 hours after transfection. For GFP expression analysis cells were analyzed directly. For surface marker analysis cells were stained with fluorophore labeled antibodies for 30 minutes in dark. The antibodies used were PE conjugated mouse anti-human CD279 (PD1, clone EH12.1) antibody (BD Biosciences, San Jose, CA) and APC conjugated mouse anti-human β2-microglobulin (B2M, clone 2M2) antibody (Biolegend, San Diego, CA).

### T7EN1 cleavage assay and sequencing

Cells were harvested 48 hours after transfection and genomic DNA was extracted with a Wizard genomic DNA purification kit (Promega, Madison, WI). Targeted regions were PCR amplified using high-fedelity PCR master mix (ThermoFisher) with primers listed in Supplemental Table 3. The products were gel purified using Qiaquick gel purification kit (Qiagen, Germantown, MD). For T7EN1 assay the purified PCR products were denatured and re-annealed and digested with T7EN1 enzyme (NEB, Ipswich, MA) for 30 minutes at 37 °C, and then analyzed by 2% agarose gel. Gene modification efficiency was calculated by % gene modification = 100 × (1 − (1 − fraction cleaved)^1/2^)^37^. DNA sequencing was used to further confirm the gene editing rate. The PCR products of the targeted regions were produced from the genomic DNA of the bulk transfected cells as described above. After purification they were ligated into a pJET1.2 vector using the CloneJET PCR cloning kit (ThermoFisher). The ligated DNA was transformed into *E.coli* and 20 to 60 colonies were picked to extract the plasmid DNA. The inserts were then sequenced using the kit supplied universal sequencing primer. We assume that the Indel rates among colonies were identical to that among the genomic DNAs.

### Cell viability assay

Cell viability was analyzed 24 hours after transfection by trypan blue dye exclusion method using Vi-Cell XR instrument (Beckman Coulter, Indianapolis, IN). Adherent cells were dissociated to single cells using the enzyme indicated in Cell Culture. Since dead cells are often floating the cell culture medium was centrifuged and the floating cells were combined with the dissociated cells for cell count. Cells were put in the sample tube, trypan blue mixing and viable cell count were done automatically by the instrument.

### gRNA synthesis

All primers were ordered from IDT (Coralville, Iowa). Primer pairs were annealed to PCR assemble the gRNA DNA template, and then synthesize the gRNA by *in vitro* transcription using GeneArt Precision gRNA synthesis kit (ThermoFisher). The gRNA was then purified using the GeneJET RNA purification column supplied with the kit and the concentration measured by Nanodrop (ThermoFisher).

### Western Blot analysis

Cells were harvested 48 hours after transfection and lysed with RIPA lysis and extraction buffer (ThermoFisher). The protein concentration was determined using Pierce BCA protein assay kit (ThermoFisher). The cell lysate was fractionated using a 4–12% Mini-PROTEAN TGX gel (Bio-Rad (Hercules, CA) and transferred to a PVDF membrane. Upon blocking, the membrane was incubated for 2 h with polyclonal rabbit GFP antibody at 1:1000 dilution. After washing, the membrane was incubated for 1 hour with HRP conjugated goat anti-rabbit antibody at 1:2000 dilution. Upon extensive washing, the membrane was developed with Pierce ECL reagent (ThermoFisher) and imaged using a Fuji imager LAS 4000 instrument (GE, Pittsburgh, PA).

### Statistical analysis

Microsoft Excel (Seattle, WA) was used for all statistical analysis. The mean ± S.E.M. was determined for each treatment group in the individual experiments. And the one-tailed Student t-test was used to determine the significances between treatment and control group. P-values < 0.05 were considered significant.

## Electronic supplementary material

Supplementary information
