# Supplementary material for: Efficient homology-directed gene editing by CRISPR/Cas9 in human stem and primary cells using tube electroporation
Source: Sci Rep. 2018 Aug 3;8:11649. doi: 10.1038/s41598-018-30227-w (PMC6076306; doi:10.1038/s41598-018-30227-w)
Supplement: Supplementary file 1 — Supplementary information [file 41598_2018_30227_MOESM1_ESM.pdf]

Supplemental information

Title: Efficient homology-directed gene editing by CRISPR/Cas9 in human stem and primary cells using tube electroporation

Xiaoyun Xu[1, †], Dongbing Gao[1, †], Ping Wang[2], Jian Chen[3], Jinxue Ruan[4], Jie Xu[4, *], Xiaofeng Xia[1, 5, *]

[1]Chao Center for BRAIN, Department of Systems Medicine and Bioengineering, Houston Methodist Research Institute, Houston, Texas, USA

[2]Department of Pathology and Laboratory Medicine, University of Pennsylvania Perelman School of Medicine, Philadelphia, Pennsylvania, USA

[3]Celetrix Biotechnologies, Manassas, Virginia, USA

[4]Center for Advanced Models and Translational Sciences and Therapeutics, University of Michigan Medical School, Ann Arbor, MI 48109-2800

[5]Weill Cornell Medical College, Cornell University, New York, New York, USA

[†]These authors contributed equally to this work

*Correspondence and requests for materials should be addressed to X.X. (email: xiaofengxia01@gmail.com) or J.X. (email: jiex@umich.edu).

Supplemental Table 1. Electroporation conditions for different cell types

Cell Type	Tube size (μl)	Voltage (V)	Pulse time (ms)
**DNA transfection (30 nM pCMV-GFP)**			
Human iPSC	120	640	30 × 1
HEK293 cell	120	620	30 × 1
Mouse iPSC	120	660	30 × 1
Jurkat cell	120	660	30 × 1
Human adipose MSC	20	440	30 × 1
Human neural progenitor cell ReNCell VM	120	620	30 × 1
Human primary T cell	20	440	30 × 1
Human cord blood MSC	120	640	30 × 1
Rat primary cortex neural progenitor cell	120	620	30 × 1
Human ESC derived neural stem cell	120	640	30 × 1
Mouse ESC	120	660	30 × 1
Human primary hepatocyte	20	440	30 × 1
Human MM1.S	20	535 + 315	5 × 1 + 60 × 1
Human bone marrow MSC	20	440	30 × 1
**Protein transfection (0.5 μM Alexa Fluor 488-IgG)**			
Human iPSC	120	620	30 × 1
Human primary T cell	20	415	30 × 1
Human adipose MSC	20	415	30 × 1
**dsRNA transfection (0.5 μM FAM-21-mer-dsRNA)**			
Human iPSC	120	620	30 × 1
Human primary T cell	20	415	30 × 1
Human adipose MSC	20	415	30 × 1
**Cas9/gRNA RNP transfection (0.5 μM Cas9-NLS, 0.85 μM gRNA, with or without 2.7 μM ssODN)**			
Human iPSC	120	620	30 × 1
HEK293 cell	120	620	30 × 1
Human MSC	120	620	30 × 1
Human primary T cell	120	660	30 × 1

Supplemental Table 2. Comparison with other electroporation instruments. Cells were transfected with pCMV-GFP plasmid and the transfection efficiencies were measured by flow cytometry analysis of GFP expression rates. The actual survival rates were listed on the top. The relative survival rates compared to the untransfected controls were given in parenthesis at the bottom. The survival rates for the control untransfected cells during regular passage were 83.7±4.4; 90.1±3.6; 93.3±0.7 and 93.4±2.9 for human iPSC, Jurkat, mouse ESC and ReNCell VM cells. The electroporation conditions for Bio-Rad Gene Pulser Xcell and Lonza 4D Nucleofector were listed under each cell type. The tube electroporation conditions were listed in Supplemental Table 1.

	human iPSC		Jurkat		Mouse ESC		ReNCell VM	
	Efficiency	Survival	Efficiency	Survival	Efficiency	Survival	Efficiency	Survival
Tube electroporation	74.0±4.9%	73.1±5.3% (87.3%)	75.5±2.3%	73.2±5.5% (81.2%)	85.2±3.7%	92.1±3.1% (98.7%)	83.1±3.5%	93.6±3.0% (100.2%)
Bio-Rad Gene Pulser Xcell	15.1±2.1%	24.8±4.5% (29.6%)	7.5±2.7%	46.0±9.1% (51.1%)	56.6±6.3%	58.7±1.6% (62.9%)	25.7±1.8%	43.9±1.7% (47.0%)
	250V, 500µF, 1000Ω		300V, 350µF, 1000Ω		795V, 10µF, [illegible]Ω, 0.2ms		250V, 500µF, 1000Ω	
Lonza 4D Nucleofector	46.5±3.8%	55.5±1.7% (66.3%)	44.4±10.9%	57.9±3.0% (64.3%)	74.4±3.7%	76.3±6.6% (81.8%)	45.0±4.0%	63.1±6.1% (67.6%)
	P3 solution, CB-150 program		V4XC solution, CL-120 program		P3 solution, CG-104 program		SG solution, DN-100 program	

Supplemental Table 3. DNA sequences and primers for genome editing

**Target sequences (PAMs are shaded and not included)**		
Gene	gRNA#	
PDCD1	gRNA1	GGCGTGACTTCCACATGAGCGTGG
	gRNA2	GGCAGTTGTGTGACACGGAAGCGG
	gRNA3	TGACGTTACCTCGTGCGGCCCGG
APP	gRNA1	GCAGAATTCCGACATGACTCAGG
B2M	gRNA4	CCAGAAAGAGAGAGTAGCGCGAG
	gRNA6	GGCCGAGATGTCTCGCTCCGTGG
	gRNA8	CCACCTCTTGATGGGGCTAGTAG
	gRNA13	GCTACTCTCTCTTTCTGGCCTGG
AAVS1	gRNA1	GCCAGTAGCCAGCCCCGTCCTGG
OCT4	gRNA1	TCTCCCATGCATTCAAACTGAGG

**Primers for gRNA synthesis**			
Gene	gRNA#		
PDCD1	gRNA1	Fwd	TAATACGACTCACTATAGGCGTGACTTCCACATG
		Rev	TTCTAGCTCTAAAACCGCTCATGTGGAAGTCACG
	gRNA2	Fwd	TAATACGACTCACTATAGCAGTTGTGTGACACGG
		Rev	TTCTAGCTCTAAAACCTTCCGTGTCACACAACTG
	gRNA3	Fwd	TAATACGACTCACTATAGTGACGTTACCTCGTGC
		Rev	TTCTAGCTCTAAAACGGCCGCACGAGGTAACGTC
APP	gRNA1	Fwd	TAATACGACTCACTATAGCAGAATTCCGACATGA
		Rev	TTCTAGCTCTAAAACGAGTCATGTCGGAATTCTG
B2M	gRNA4	Fwd	TAATACGACTCACTATAGCCAGAAAGAGAGAGTA
		Rev	TTCTAGCTCTAAAACGCGCTACTCTCTCTTTCTG
	gRNA6	Fwd	TAATACGACTCACTATAGGCCGAGATGTCTCGCT
		Rev	TTCTAGCTCTAAAACCGGAGCGAGACATCTCGGC
	gRNA8	Fwd	TAATACGACTCACTATAGCCACCTCTTGATGGGG
		Rev	TTCTAGCTCTAAAACCTAGCCCCATCAAGAGGTG
	gRNA13	Fwd	TAATACGACTCACTATAGCTACTCTCTCTTTCTG
		Rev	TTCTAGCTCTAAAACGGCCAGAAAGAGAGAGTAG
AAVS1	gRNA1	Fwd	TAATACGACTCACTATAGGCCAGTAGCCAGCCCC
		Rev	TTCTAGCTCTAAAACGGACGGGGCTGGCTACTGG
OCT4	gRNA1	Fwd	TAATACGACTCACTATAGTCTCCCATGCATTCAA
		Rev	TTCTAGCTCTAAAACCAGTTTGAATGCATGGGAG

Primers for PCR amplifying the target regions			
PDCD1	gRNA1	Fwd	GGACAACGCCACCTTCACCTGC
		Rev	CTACGACCCTGGAGCTCCTGAT
	gRNA2	Fwd	GGACAACGCCACCTTCACCTGC
		Rev	CTACGACCCTGGAGCTCCTGAT
	gRNA3	Fwd	TCCTAACCCCTGACCTTTGTGC
		Rev	CGGTGCGCCTGGCTCCTATTGT
APP	gRNA1	Fwd	GGGTAGGCTTTGTCTTACAGTGTTAT
		Rev	ATCCTATAGGCAAGCATTGTATTTTTA
B2M	gRNA4	Fwd	CTGGCTTGGAGACAGGTGAC
		Rev	GACGCTTATCGACGCCCTAA
	gRNA6	Fwd	CTGGCTTGGAGACAGGTGAC
		Rev	GACGCTTATCGACGCCCTAA
	gRNA8	Fwd	CAAAATCTTGCCGCCTTCCC
		Rev	ACTTTCCAAAATGAGAGGCATGA
	gRNA13	Fwd	CTGGCTTGGAGACAGGTGAC
		Rev	GACGCTTATCGACGCCCTAA
AAVS1	gRNA1	Fwd	GAATATGTCCCAGATAGCAC
		Rev	GTTCTCAGTGGCCACCCTGC
OCT4	gRNA1	Fwd	CAGATCAGCCACATCGCCCA
		Rev	ATCAAGAACATCATTGAACT

ssODNs		
Gene	gRNA#	
PDCD1	gRNA2	TGACCACGCTCATGTGGAAGTCACGCCCGTTGGGCAGTTGTGTGACACGGAAGCGCAGTCCTGGCCGGGCTGGCTGCGGTCCTCGGGGAAGGCGGCCAG
APP	gRNA1	AAGACGGAGGAGATCTCTGAAGTGAATCTGGATGCAGAATTCCGACATGACTCAGCATATGAAGTTCATCATCAAAAATTGGTACGTAAAATAATTTACC
B2M	gRNA4	GGGTAGGAGAGACTCACGCTGGATAGCCTCCAGGCCAGAAAGAGAGAGTAGCGCGACGCACAGCTAAGGCCACGGAGCGAGACATCTCGGCCCGAATGCT
	gRNA6	GCTGGCGGGCATTCCTGAAGCTGACAGCATTCGGGCCGAGATGTCTCGCTCCGTGCGCCTTAGCTGTGCTCGCGCTACTCTCTCTTTCTGGCCTGGAGGC

Supplemental Table 4-1.DNA sequencing results of human iPSC transfected with RNP targeting APP gene. Mutated sites are in red.

WT	GAAGATGGATGCAGAATTCCGAC---------ATGA-CTCAGGATATGAAGTT
**Experiment Batch 1**	
Clone#1.	GAAGATGGGTGCAGAA--CCGAC---------ATGA-CTCAGGATATGAAGTT
Clone#2.	GAAGATGGATGCAGAATTCCGAC---------ATGA-CTCAGGATATGAAGTT
Clone#3.	GAAGATGGATGCAGAATTCCGAC---------ATGAACTCAGGATATGAAGTT
Clone#4.	GAAGATGGATGCAGAATTCCGAC---------ATGA-CTCAGGATATGAAGTT
Clone#6.	GAAGATGGATGCAGAATTCCGAC---------ATGA-CTCAGGATATGAAGTT
Clone#7.	GAAGATGGATGCAGAATTCCGAT--------------------ATATGAAGTT
Clone#8.	GAAGATGGATGCAGAATTCCGAC---------ATGA-CTCAGGATATGAAGTT
Clone#9.	GAAGATGGATGCAGAATTCCGACCTCGATTATGTCCCTTCAGGATATGAAGTT
Clone#10.	GAAGATGGATGCAGAATTCCGAC---------ATGA-----GGATATGAAGTT
**Experiment Batch 2**	
Clone#11.	GAAGATGGATGCAGAATTCCGAC---------ATGA-CTCAGGATATGAAGTT
Clone#13.	GAAGATGGATGCAGAATTCCGAC---------ATGAACTCAGGATATGAAGTT
Clone#14.	GAAGATGGATGCAGAATTCCGAC---------ATGA-CTCAGGATATGAAGTT
Clone#15.	GAAGATGGATGCAGAATTCCGAC---------ATGA-------ATATGAAGTT
Clone#16.	GAAGATGGATGCAGAATTCCGAC---------ATGA-CTCAGGATATGAAGTT
Clone#17.	GAAGATGGATGCAGAATTCCGAC---------ATGA--ACAGGATATGAAGTT
Clone#18.	GAAGATGGATGCAGAATTCCGAC---------ATGA--------TATGAAGTT

Total clones sequenced 16	
Wildtype 43.8%	Indels 56.3%

Supplemental Table 4-2.DNA sequencing results of human iPSC transfected with RNP targeting APP gene and ssODN template carrying G to C mutation in PAM. Mutated sites are in red.

WT	GAAGATGGATGCAGAATTCCGAC--------------------------------ATGA-CTCAGGATATGAAGTT

Experiment Batch 1

Clone#1.	GAAGATGGGTGCAGAATTCCGAC--------------------------------ACGA---CAGGATATGAAGTT
Clone#2.	GAAGATGGATGCAGAATTCCGAC--------------------------------ATGA-CTCAGGATATGAAGTT
Clone#3.	GAAGATGGATGCAGAATTCCGAC--------------------------------ATGA-CTCAGCATATGAAGTT
Clone#4.	GAAGATGGATGCAGAATTCCGAC--------------------------------ATGA-CTCAGCATATGAAGTT
Clone#5.	GAAGATGGATGCAGAATTCCGAC--------------------------------ATGAACTCAGGATATGAAGTT
Clone#6.	GAAGATGGATGCAGAATTCCGAC--------------------------------ATGA-----------GAAGTT
Clone#7.	GAAGATGGATGCAGAATTCCGAC--------------------------------ATGAACTCAGGATATGAAGTT
Clone#8.	GAAGATGGATGCAGAATTCCGA--------------------------ATTGAATCATC-------ATATGAAGTT
Clone#10.	GAAGATGGATGCAGAATTCCGAC--------------------------------ATGA-CTCAGGATATGAAGTT
Clone#11.	GAAGATGGATGCAGAATTCCGAC--------------------------------ATGA-CTCAGCATATGAAGTT
Clone#12.	GAAGATGGATGCAGAATTCCGAC--------------------------------ATGA-CTCAGCATATGAAGTT
Clone#13.	GAAGATGGATGCAGAATTCCGAC--------------------------------ATGA-CTCAGGATATGAAGTT
Clone#14.	GAAGATGGATGCAGAATTCGGCTTGGGTAGGCTTTGTCTTACAGTGTTATTATTTATGA-CTCAGCATATGAAGTT
Clone#15.	GAAGATGGATGCAGAATTCCGAC--------------------------------ATGA--------TATGAAGTT
Clone#16.	GAAGATGGATGCAGAATTCCGAC--------------------------------ATGA-CTCAGCATATGAAGTT
Clone#17.	GAATCTGGATGCAGAATTCCGAC--------------------------------ATGA-CTCAGCATATGAAGTT
Clone#18.	GAAGATGGATGCGGAATTCCGAC--------------------------------ATGA--TCAGCATATGAAGTT
Clone#19.	GAAGATGGATGCAGAATTCCGAC--------------------------------ATGA-CTCAGCATATGAAGTT

Experiment Batch 2

Clone#20.	GAAGATGGATGCAGAATTCCGAC--------------------------------AT---CTCAGGATATGAAGTT
Clone#21.	GAAGATGGATGCAGAATTCCGAC--------------------------------ATGA-CTCAGCATATGAAGTT
Clone#22.	GAAGATGGATGCAGAATTCCGAC--------------------------------ATGA-CTCAGCATATGAAGTT
Clone#23.	GAAGATGGATGCAGAATTCCGAC--------------------------------ATGAACTCAGGATATGAAGTT
Clone#24.	GAAGATGGATGCAGAATTCCGAC--------------------------------ATG---TCAGGATATGAAGTT
Clone#26.	GAAGATGGATGCAGAATTCCGAC--------------------------------ATGA-------ATATGAAGTT
Clone#27.	GAAGATGGATGCAGAATTCCGAC--------------------------------ATGAACTCAGGATATGAAGTT
Clone#28.	GAAGATGGATGCAGAATTCCGAC--------------------------------ATGA-CTCAGCATATGAAGTT
Clone#29.	GAAGATGGATGCAGAATTCCGAC--------------------------------ATGA-CTCAGCATATGAAGTT
Clone#30.	GAAGATGGATGCAGAATTCCGAC--------------------------------ATGA-CTCAGCATATGAAGTT
Clone#31.	GAAGATGGATGCAGAATTCCGAC--------------------------------ATGA--ACAGGATATGAAGTT
Clone#32.	GAAGATGGATGCAGAATTCCGAC--------------------------------ATGA--------TATGAAGTT
Clone#33.	GAATCTGGATGCAGAATTCCGAC--------------------------------ATGAACTCAGGATATGAAGTT
Clone#34.	GAAGATGGATGCAGAATTCCGAC--------------------------------ATGA-CTCAGCATATGAAGTT

```
Clone#35.   GAAGATGGATGCAGAATTCCGAC--------------------------------ATGA-CTCAGCATATGAAGTT
Clone#38.   GAAGATGGATGCAGAATTCCGAC-------------------------------------TTCAGGATATGAAGTT
Clone#39.   GAAGATGGATGCAGAATTCCGAC--------------------------------ATGA-CTCAGCATATGAAGTT
Clone#40.   GAAGATGGATGCAGAATTCCGAC--------------------------------ATGA-CTCAGCATATGAAGTT
Clone#41.   GAAGATGGATGCAGAACTCAAG---------------------------------------TCAGCATATGAAGTT
Clone#43.   GAAGATGGATGCAGAATTCCGAC--------------------------------ATGA-CTCAGGATATGAAGTT
```

<table>
<tr><td colspan="3">Total clones sequenced
38</td></tr>
<tr><td rowspan="2">Wildtype
10.5%</td><td colspan="2">Mutations
89.5%</td></tr>
<tr><td>InDels
47.4%</td><td>Scarless point (G→C) mutations
42.1%</td></tr>
</table>

Supplemental table 5. Pathological mutations that eliminate or create PAM

A. Summary

Total gene number 22	
Total mutation number 3,382	
Mutations eliminating PAM 526 (15.6%)	Mutations creating PAM 322 (9.5%)

B. Full mutation list

	HGMD accession#	DNA mutation	Amino acid change	phenotype
**Gene name:ATP7B** mutations eliminate a PAM (78)	CM980163	GGC-GTC	Gly-Val	Wilson disease
	CM123052	GGA-GTA	Gly-Val	Wilson disease
	HM050020	GGT-GTT	Gly-Val	Wilson disease
	CM128750	CCC-CTC	Pro-Leu	Wilson disease
	CM053128	GGC-GAC	Gly-Asp	Wilson disease
	CM950109	GGC-GCC	Gly-Ala	Wilson disease
	CM074060	TCC-TAC	Ser-Tyr	Wilson disease
	CM973225	TCC-TGC	Ser-Cys	Wilson disease
	CM960121	GGT-CGT	Gly-Arg	Wilson disease
	CM983395	GGT-GCT	Gly-Ala	Wilson disease
	CM053130	GGT-GTT	Gly-Val	Wilson disease
	CM960122	GGG-GAG	Gly-Glu	Wilson disease
	CM992593	GGG-TGG	Gly-Trp	Wilson disease
	CM1210111	GCC-GAC	Ala-Asp	Wilson disease
	CM1210110	GCC-GTC	Ala-Val	Wilson disease
	CM125004	GCC-GTC	Ala-Val	Wilson disease
	CM016106	AGG-ATG	Arg-Met	Wilson disease
	CM024203	CCC-CAC	Pro-His	Wilson disease
	CM067648	CCC-CTC	Pro-Leu	Wilson disease
	CM950111	CGG-CTG	Arg-Leu	Wilson disease
	CM053796	TGG-TGA	Trp-Term	Wilson disease
	CM1111074	ACC-ATC	Thr-Ile	Wilson disease
	CM128748	AGG-AGT	Arg-Ser	Wilson disease
	CM076005	CGG-CCG	Arg-Pro	Wilson disease
	CM123056	GGG-GAG	Gly-Glu	Wilson disease
	CM111988	ACC-ATC	Thr-Ile	Wilson disease
	CM117982	TCC-TAC	Ser-Tyr	Wilson disease
	CM962394	GGA-GTA	Gly-Val	Wilson disease

CM074057	GGC-GAC	Gly-Asp	Wilson disease
CM960127	GGC-GTC	Gly-Val	Wilson disease
CM119350	TGG-TGT	Trp-Cys	wilson diseasewith early and/or severe hepatic disease
CM980179	GGT-GAT	Gly-Asp	Wilson disease
CM994112	GGT-TGT	Gly-Cys	Wilson disease
CM032850	TCC-TAC	Ser-Tyr	Wilson disease
CM076007	GCC-GTC	Ala-Val	Wilson disease
CM107540	TCC-TTC	Ser-Phe	Wilson disease
CM1210113	GGG-GTG	Gly-Val	Wilson disease
CM051387	CCC-CAC	Pro-His	Wilson disease
CM114901	GGC-GAC	Gly-Asp	Wilson disease
CM075999	GGC-CGC	Gly-Arg	Wilson disease
CM076008	GGC-GTC	Gly-Val	Wilson disease
CM110340	CCC-CTC	Pro-Leu	Wilson disease
CM053785	ACC-AGC	Thr-Ser	Wilson disease
CM970143	GGC-GTC	Gly-Val	Wilson disease
CM992820	CGG-CCG	Arg-Pro	Wilson disease
CM992599	CCC-CTC	Pro-Leu	Wilson disease
CM992600	GGG-GAG	Gly-Glu	Wilson disease
CM960130	GGA-GAA	Gly-Glu	Wilson disease
CM980184	GGA-GTA	Gly-Val	Wilson disease
CM032851	CCA-CGA	Pro-Arg	Wilson disease
CM024365	GGC-GAC	Gly-Asp	Wilson disease
CM095180	GGC-GCC	Gly-Ala	Hepatocerebral degeneration
CM110342	GGA-GAA	Gly-Glu	Wilson disease
CM081179	GGA-GCA	Gly-Ala	Wilson disease
CM980187	TGG-TGT	Trp-Cys	Wilson disease
CM116729	GGT-GTT	Gly-Val	Wilson disease
CM053127	GGA-GAA	Gly-Glu	Wilson disease
CM970146	GGT-TGT	Gly-Cys	Wilson disease
CM980191	GGT-GTT	Gly-Val	Wilson disease
CM053129	GGG-GAG	Gly-Glu	Wilson disease
CM1210112	CCT-ACT	Pro-Thr	Wilson disease
CM970148	GGG-GTG	Gly-Val	Wilson disease
CM061645	CCG-CAG	Pro-Gln	Wilson disease
CM035477	CCG-TCG	Pro-Ser	Wilson disease
CM074058	GGT-GAT	Gly-Asp	Wilson disease
CM080090	GGT-TGT	Gly-Cys	Wilson disease
CM125013	GGC-CGC	Gly-Arg	Wilson disease
CM118182	GCC-GAC	Ala-Asp	Wilson disease
CM080086	AGG-AGC	Arg-Ser	Wilson disease

	CM082493	GGT-CGT	Gly-Arg	Wilson disease
	CM980194	GGT-GAT	Gly-Asp	Wilson disease
	CM053131	GGT-GTT	Gly-Val	Wilson disease
	CM076002	CCC-CGC	Pro-Arg	Wilson disease
	CM057647	CCC-CTC	Pro-Leu	Wilson disease
	CM107542	GGC-GAC	Gly-Asp	Wilson disease
	CM053791	GGC-TGC	Gly-Cys	Wilson disease
	CM053125	CCT-TCT	Pro-Ser	Wilson disease
	CM082497	TGG-TGA	Trp-Term	Wilson disease
mutations create a PAM (45)	CM077234	GAC-GGC	Asp-Gly	wilson disease
	CM129457	GCA-CCA	Ala-Pro	wilson disease
	CM125002	GAG-GGG	Glu-Gly	Wilson disease
	CM081181	GTC-GCC	Val-Ala	Wilson disease
	CM970138	ATG-AGG	Met-Arg	Wilson disease
	CM970139	CTC-CCC	Leu-Pro	Wilson disease
	CM022324	TCT-CCT	Ser-Pro	Wilson disease
	CM992594	TCT-CCT	Ser-Pro	Wilson disease
	CM053126	GCG-GGG	Ala-Gly	Wilson disease
	CM032847	GAC-GGC	Asp-Gly	Wilson disease
	CM045739	ACG-AGG	Thr-Arg	Wilson disease
	CM1210109	ACG-CCG	Thr-Pro	wilson disease
	CM992595	ATG-AGG	Met-Arg	Wilson disease
	CM109468	CTC-CCC	Leu-Pro	Wilson disease
	CM994110	GAA-GGA	Glu-Gly	Wilson disease
	CM950113	ATC-ACC	Ile-Thr	Wilson disease
	CM125006	GAA-GGA	Glu-Gly	Wilson disease
	CM123057	GTC-GGC	Val-Gly	Wilson disease
	CM110338	GCG-CCG	Ala-Pro	Wilson disease
	CM072876	GAC-GCC	Asp-Ala	Wilson disease
	CM980178	ACT-CCT	Thr-Pro	Wilson disease
	CM040681	GTT-GGT	Val-Gly	Wilson disease
	CM111989	ACG-AGG	Thr-Arg	Wilson disease
	CM081172	CAG-CCG	Gln-Pro	Wilson disease
	CM080087	ATG-AGG	Met-Arg	Wilson disease
	CM068336	CAT-CCT	His-Pro	Wilson disease
	CM962395	CTC-CCC	Leu-Pro	Wilson disease
	CM992822	GAA-GGA	Glu-Gly	Wilson disease
	CM992601	CAG-CCG	Gln-Pro	Wilson disease
	CM044579	GTC-GCC	Val-Ala	Wilson disease
	CM061648	GCT-CCT	Ala-Pro	Wilson disease
	CM980188	GCT-GGT	Ala-Gly	Wilson disease

	CM061647	GCA-CCA	Ala-Pro	Wilson disease
	CM1210108	CAG-CCG	Gln-Pro	Wilson disease
	CM125010	CAG-CCG	Gln-Pro	Wilson disease
	CM057645	ATC-ACC	Ile-Thr	Wilson disease
	CM992602	GTC-GGC	Val-Gly	Wilson disease
	CM125012	GCA-GGA	Ala-Gly	Wilson disease
	CM003413	GAC-GGC	Asp-Gly	Wilson disease
	CM064991	ACG-AGG	Thr-Arg	Wilson disease
	CM970150	CGC-CCC	Arg-Pro	Wilson disease
	CM053788	CTC-CCC	Leu-Pro	Wilson disease
	CM001617	CTC-CCC	Leu-Pro	Wilson disease
	CM053133	TGC-TCC	Cys-Ser	Wilson disease
	CM107544	GCA-CCA	Ala-Pro	Wilson disease
**Gene name:CP**				
mutations eliminate a PAM (5)	CM035016	CCT-CGT	Pro-Arg	Aceruloplasminaemia
	CM066770	TGG-TCG	Trp-Ser	Aceruloplasminaemia
	CM057327	GCC-GAC	Ala-Asp	Aceruloplasminaemia
	CM035041	GGA-GAA	Gly-Glu	Multiple sytem atrophy
	CM066769	GGC-GCC	Gly-Ala	Aceruloplasminaemia
**Gene name:HEXA**				
mutations eliminate a PAM (11)	CM910214	TGG-TGA	Trp-Term	Tay-Sachs disease
	CM910217	TCC-TTC	Ser-Phe	Tay-Sachs disease
	CM970717	TCC-TTC	Ser-Phe	Tay-Sachs disease
	CM920336	GGT-GAT	Gly-Asp	Tay-Sachs disease
	CM001188	GGT-GTT	Gly-Val	Tay-Sachs disease
	CM126852	CCT-TCT	Pro-Ser	Tay-Sachs disease
	CM970718	GGT-GAT	Gly-Asp	Tay-Sachs disease
	CM900127	TGG-TGC	Trp-Cys	Tay-Sachs disease
	CM034664	TGG-TGT	Trp-Cys	Gangliosidosis GM2
	CM983745	GGT-GAT	Gly-Asp	Tay-Sachs disease
	CM980972	TGG-TGC	Trp-Cys	Gangliosidosis GM2
mutations create a PAM (5)	CM952144	CGC-GGC	Arg-Gly	Tay-Sachs disease
	CM930395	TTC-TCC	Phe-Ser	Tay-Sachs disease
	CM970719	TCT-CCT	Ser-Pro	Tay-Sachs disease
	CM930397	ATG-AGG	Met-Arg	Tay-Sachs disease
	CM125500	CGA-CCA	Arg-Pro	Tay-Sachs disease
**Gene**				

**name:PPOX**				
mutations eliminate a PAM (16)	CM0911069	GGA-TGA	Gly-Term	Porphyria, variegate
	CM066970	GGC-GAC	Gly-Asp	Porphyria, variegate
	CM992403	GGA-GAA	Gly-Glu	Porphyria, variegate
	CM040243	GGA-GCA	Gly-Ala	Porphyria, variegate
	CM030943	TGG-TGA	Trp-Term	Porphyria, variegate
	CM001311	CGG-CCG	Arg-Pro	Porphyria, variegate
	HM070048	GGC-GAC	Gly-Asp	Porphyria, variegate
	CM981617	GGA-GAA	Gly-Glu	Porphyria, variegate
	CM992413	TGG-TGA	Trp-Term	Porphyria, variegate
	CM961147	GGT-CGT	Gly-Arg	Porphyria, variegate
	CM011458	CCG-CGG	Pro-Arg	Reduced activity
	CM031720	GGA-CGA	Gly-Arg	Porphyria, variegate
	CM087304	GGA-GCA	Gly-Ala	Porphyria, variegate
	CM992419	TGG-TGA	Trp-Term	Porphyria, variegate
	CM992420	TGG-TGA	Trp-Term	Porphyria, variegate
	CM992423	GGA-GTA	Gly-Val	Porphyria, variegate
mutations create a PAM (11)	CM011457	ATC-ACC	Ile-Thr	Porphyria, variegate
	CM961144	CAC-CCC	His-Pro	Porphyria, variegate
	CM992402	CGT-CCT	Arg-Pro	Porphyria, variegate
	CM992404	CTC-CCC	Leu-Pro	Porphyria, variegate
	CM992405	GTG-GGG	Val-Gly	Porphyria, variegate
	CM003896	CAT-CCT	His-Pro	Porphyria, variegate
	CM992416	CTC-CCC	Leu-Pro	Porphyria, variegate
	CM992417	GTG-GGG	Val-Gly	Porphyria, variegate
	CM981618	GAC-GCC	Asp-Ala	Porphyria, variegate
	CM992418	TCA-CCA	Ser-Pro	Porphyria, variegate
	CM961148	GCT-CCT	Ala-Pro	Porphyria, variegate
**Gene name:HBB**				
mutations eliminate a PAM (61)	CM840028	CCT-CGT	Pro-Arg	Haemoglobin variant
	CM031994	CCT-GCT	Pro-Ala	Haemoglobin variant
	CM940915	CCT-TCT	Pro-Ser	Haemoglobin variant
	CM860058	GCC-GAC	Ala-Asp	Haemoglobin variant
	CM990717	GCC-GTC	Ala-Val	Haemoglobin variant
	CM092642	GCC-GTC	Ala-Val	Thalassaemia beta
	CM900120	TGG-TGA	Trp-Term	Thalassaemia beta
	CM087631	GGC-CGC	Gly-Arg	Haemoglobin variant
	CM860029	GGC-GAC	Gly-Asp	Haemoglobin variant
	CM870088	GGT-GAT	Gly-Asp	Haemoglobin variant

CM930370	GCC-GAC	Ala-Asp	Haemoglobin variant
CM973679	GCC-GGC	Ala-Gly	Haemoglobin variant
CM952029	GGC-GAC	Gly-Asp	Haemoglobin variant
CM930372	AGG-AGT	Arg-Ser	Haemoglobin variant
CM870034	CCT-ACT	Pro-Thr	Haemoglobin variant
CM001710	CCT-CAT	Pro-His	Haemoglobin variant
CM880151	CCT-CGT	Pro-Arg	Haemoglobin variant
CM993614	CCT-GCT	Pro-Ala	Haemoglobin variant
CM850014	CCT-TCT	Pro-Ser	Haemoglobin variant
CM830030	TGG-TCG	Trp-Ser	Haemoglobin variant
CM860011	TGG-TGA	Trp-Term	Thalassaemia beta
CM1110588	TGG-TGT	Trp-Cys	Haemoglobin variant
CM962426	ACC-AAC	Thr-Asn	Haemoglobin variant
CM066097	ACC-ATC	Thr-Ile	Haemoglobin variant
CM870035	TCC-TGC	Ser-Cys	Haemoglobin variant
CM931407	GGG-GAG	Gly-Glu	Haemoglobin variant
CM880152	TCC-TTC	Ser-Phe	Haemoglobin variant
CM830029	CCT-CGT	Pro-Arg	Haemoglobin variant
BM1132884	GGC-CGC	Gly-Arg	Thalassaemia beta
CM920320	GGC-GAC	Gly-Asp	Haemoglobin variant
CM076229	GGC-TGC	Gly-Cys	Haemoglobin variant
CM952452	CCT-CGT	Pro-Arg	Haemoglobin variant
CM024479	GGC-GAC	Gly-Asp	Haemoglobin variant
CM024478	GGC-GCC	Gly-Ala	Haemoglobin variant
CM942304	GCC-GAC	Ala-Asp	Haemolytic anaemia
CM057104	GCC-GGC	Ala-Gly	Thalassaemia beta
CM066099	GCC-GTC	Ala-Val	Haemoglobin variant
CM810022	GGC-GAC	Gly-Asp	Haemoglobin variant
CM950616	GGC-GTC	Gly-Val	Haemolytic anaemia
CM940920	GGC-GAC	Gly-Asp	Haemoglobin variant
CM0910548	ACC-AAC	Thr-Asn	Haemoglobin variant
CM870036	GCC-GAC	Ala-Asp	Haemoglobin variant
CM128485	GCC-GTC	Ala-Val	Haemoglobin variant
CM850041	CCT-CGT	Pro-Arg	Haemoglobin variant
CM099802	CCT-GCT	Pro-Ala	Haemoglobin variant
CM920323	AGG-ACG	Arg-Thr	Haemoglobin variant
CM044639	AGG-AGC	Arg-Ser	Haemoglobin variant
CM066874	GGC-GAC	Gly-Asp	Haemoglobin variant
CM930380	GCC-GAC	Ala-Asp	Thalassaemia beta
CM097720	GCC-GTC	Ala-Val	Haemoglobin variant
CM940924	GGC-GAC	Gly-Asp	Haemoglobin variant
CM045165	GGC-GCC	Gly-Ala	Haemoglobin variant
CM014199	ACC-ATC	Thr-Ile	Haemoglobin variant
CM066870	CCA-CAA	Pro-Gln	Erythrocytosis
CM995296	CCA-CGA	Pro-Arg	Haemoglobin variant

	CM102820	GCC-GTC	Ala-Val	Thalassaemia beta
	CM024482	GGT-GAT	Gly-Asp	Haemoglobin variant
	CM860057	GCC-GAC	Ala-Asp	Haemoglobin variant
	CM950621	GCC-GTC	Ala-Val	Haemoglobin variant
	CM800018	GCC-GAC	Ala-Asp	Haemoglobin variant
	CM099825	GCC-GTC	Ala-Val	Haemoglobin variant
mutations create a PAM (48)	CM900119	ATG-AGG	Met-Arg	Thalassaemia beta
	CM860010	CAT-CCT	His-Pro	Haemoglobin variant
	CM994596	GAG-GGG	Glu-Gly	Haemoglobin variant
	CM962425	CTG-CGG	Leu-Arg	Haemoglobin variant
	CM930367	GTG-GGG	Val-Gly	Haemoglobin variant
	CM960814	GTG-GGG	Val-Gly	Haemoglobin variant
	CM810018	GAT-GGT	Asp-Gly	Haemoglobin variant
	CM810019	GTT-GGT	Val-Gly	Haemoglobin variant
	CM910202	CTG-CGG	Leu-Arg	Haemoglobin variant
	CM950611	CTG-CGG	Leu-Arg	Haemoglobin variant
	CM800015	CTG-CGG	Leu-Arg	Haemoglobin variant
	CM941956	TTC-TCC	Phe-Ser	Haemoglobin variant
	CM099696	CTG-CGG	Leu-Arg	Haemoglobin variant
	CM085461	GAT-GGT	Asp-Gly	Haemoglobin variant
	CM068517	GCT-CCT	Ala-Pro	Haemoglobin variant
	CM860055	CAT-CCT	His-Pro	Haemoglobin variant
	CM931128	GTG-GGG	Val-Gly	Haemoglobin variant
	CM910205	CTC-CCC	Leu-Pro	Haemoglobin variant
	CM800016	CTG-CGG	Leu-Arg	Haemoglobin variant
	CM910589	GCT-CCT	Ala-Pro	Haemoglobin variant
	CM087534	ACA-CCA	Thr-Pro	Thalassaemia beta
	CM870090	CTG-CGG	Leu-Arg	Haemoglobin variant
	CM850039	GAG-GGG	Glu-Gly	Haemoglobin variant
	CM950617	CTG-CGG	Leu-Arg	Haemoglobin variant
	CM994597	CAC-CCC	His-Pro	Haemoglobin variant
	CM120278	TGT-TGG	Cys-Trp	Haemoglobin variant
	CM045163	GAC-GGC	Asp-Gly	Haemoglobin variant
	CM850040	CAC-CCC	His-Pro	Haemoglobin variant
	CM920970	GTG-GGG	Val-Gly	Haemoglobin variant
	CM810024	GAT-GGT	Asp-Gly	Haemoglobin variant
	CM066872	GAG-GGG	Glu-Gly	Erythrocytosis
	CM830032	AAC-ACC	Asn-Thr	Haemoglobin variant
	CM109509	CTC-CCC	Leu-Pro	Haemoglobin variant
	CM910591	CTG-CGG	Leu-Arg	Haemoglobin variant
	CM910726	GTC-GCC	Val-Ala	Haemoglobin variant
	CM960819	TGT-TGG	Cys-Trp	Haemoglobin variant
	CM057102	CAT-CCT	His-Pro	Thalassaemia beta

	CM830033	CAC-CCC	His-Pro	Haemoglobin variant
	CM095603	TTC-TCC	Phe-Ser	Haemolytic anaemia
	CM910208	GTG-GGG	Val-Gly	Haemoglobin variant
	CM950620	CAG-CCG	Gln-Pro	Thalassaemia beta
	CM014200	GCT-CCT	Ala-Pro	Haemoglobin variant
	CM870038	CAG-CCG	Gln-Pro	Haemoglobin variant
	CM930384	GCT-CCT	Ala-Pro	Haemoglobin variant
	CM920327	GCT-CCT	Ala-Pro	Haemoglobin variant
	CM962429	CTG-CGG	Leu-Arg	Haemoglobin variant
	CM066867	CAC-CCC	His-Pro	Erythrocytosis
	CM014202	CAC-CCC	His-Leu	Haemoglobin variant
**Gene name:PKHD1** mutations eliminate a PAM (33)	CM051120	CCC-CGC	Pro-Arg	Polycystic kidney disease
	CM041053	TGG-TGA	Trp-Term	Polycystic kidney disease
	CM051122	GGG-GTG	Gly-Val	Polycystic kidney disease
	CM034265	GGC-GTC	Gly-Val	Polycystic kidney disease
	CM100562	TGG-TGA	Trp-Term	Polycystic kidney disease
	CM051125	TGG-TGA	Trp-Term	Polycystic kidney disease
	CM051126	GGT-CGT	Gly-Arg	Polycystic kidney disease
	CM100417	GGG-GAG	Gly-Glu	Polycystic kidney disease
	CM051127	GGG-GCG	Gly-Ala	Polycystic kidney disease
	CM051128	GGT-GAT	Gly-Asp	Polycystic kidney disease
	CM100407	GGT-GTT	Gly-Val	Polycystic kidney disease
	CM100570	GGC-GTC	Gly-Val	Polycystic kidney disease
	CM032310	TGG-TGT	Trp-Cys	Polycystic kidney disease
	CM051133	CCC-CGC	Pro-Arg	Polycystic kidney disease
	CM100410	CCA-CGA	Pro-Arg	Polycystic kidney disease
	CM054803	ACC-ATC	Thr-Ile	Polycystic kidney disease
	CM032318	CCT-ACT	Pro-Thr	Polycystic kidney disease
	CM051150	GGC-CGC	Gly-Arg	Polycystic kidney disease
	CM100560	CCC-CTC	Pro-Leu	Polycystic kidney disease

	CM051156	TGG-TGT	Trp-Cys	Polycystic kidney disease
	CM100415	TGG-TTG	Trp-Leu	Polycystic kidney disease
	CM034272	GGC-GAC	Gly-Asp	Polycystic kidney disease
	CM100425	GGG-GAG	Gly-Glu	Polycystic kidney disease
	CM100424	GGA-CGA	Gly-Arg	Polycystic kidney disease
	CM052344	GGA-GAA	Gly-Glu	Polycystic kidney disease
	CM051178	GGT-TGT	Gly-Cys	Polycystic kidney disease
	CM051182	GGT-GTT	Gly-Val	Polycystic kidney disease
	CM054819	TGG-TGA	Trp-Term	Polycystic kidney disease
	CM051185	AGG-ACG	Arg-Thr	Polycystic kidney disease
	CM034285	TGG-TGA	Trp-Term	Polycystic kidney disease
	CM051188	CCT-CTT	Pro-Leu	Polycystic kidney disease
	CM054812	CCA-ACA	Pro-Thr	Polycystic kidney disease
	CM034288	CCA-TCA	Pro-Ser	Polycystic kidney disease
mutations create a PAM (24)	CM051119	TTC-TCC	Phe-Ser	Polycystic kidney disease
	CM100422	ATC-ACC	Ile-Thr	Polycystic kidney disease
	CM100565	CGA-GGA	Arg-Gly	Polycystic kidney disease
	CM032308	CGA-CCA	Arg-Pro	Polycystic kidney disease
	CM100439	CAT-CCT	His-Pro	Polycystic kidney disease
	CM034266	ACT-CCT	Thr-Pro	Polycystic kidney disease
	CM051137	CAT-CCT	His-Pro	Polycystic kidney disease
	CM051138	CTC-CCC	Leu-Pro	Polycystic kidney disease
	CM020492	TGT-TGG	Cys-Trp	Polycystic kidney disease
	CM100418	GTG-GGG	Val-Gly	Polycystic kidney disease
	CM100440	GTT-GGT	Val-Gly	Polycystic kidney disease
	CM054808	CAG-CCG	Gln-Pro	Polycystic kidney disease
	CM032323	GAT-GGT	Asp-Trp	Polycystic kidney disease

	CM065390	ATC-ACC	Ile-Thr	Polycystic kidney disease
	CM034274	TGC-GGC	Cys-Gly	Polycystic kidney disease
	CM104211	GTC-GCC	Val-Ala	Polycystic kidney disease
	CM034277	CTT-CCT	Leu-Pro	Polycystic kidney disease
	CM100413	GTT-GGT	Val-Gly	Polycystic kidney disease
	CM041061	GAC-GGC	Asp-Gly	Polycystic kidney disease
	CM051172	CAT-CCT	His-Pro	Polycystic kidney disease
	CM034281	GTA-GGA	Val-Gly	Polycystic kidney disease
	CM051176	CAT-CCT	His-Pro	Polycystic kidney disease
	CM100559	GAC-GGC	Asp-Gly	Polycystic kidney disease
	CM032335	GTC-GGC	Val-Gly	Polycystic kidney disease
**Gene name:PKD1** mutations eliminate a PAM (60)	CM010372	TCC-TTC	Ser-Phe	Polycystic kidney disease 1
	CM010373	TGG-TGT	Trp-Cys	Polycystic kidney disease 1
	CM074427	GCC-GAC	Ala-Asp	Polycystic kidney disease 1
	CM020485	GGT-TGT	Gly-Cys	Polycystic kidney disease 1
	CM076424	TGG-TGA	Trp-Term	Polycystic kidney disease 1
	CM057348	TCC-TTC	Ser-Phe	Polycystic kidney disease 1
	CM1210856	GGA-TGA	Gly-Term	Polycystic kidney disease 1
	CM034562	TGG-TGA	Trp-Term	Polycystic kidney disease 1
	CM1210866	GGA-TGA	Gly-Term	Polycystic kidney disease 1
	CM1210867	TGG-TGA	Trp-Term	Polycystic kidney disease 1
	CM057350	TGG-TGA	Trp-Term	Polycystic kidney disease 1
	CM1210871	TGG-TGA	Trp-Term	Polycystic kidney disease 1
	CM076430	TGG-TGA	Trp-Term	Polycystic kidney disease 1
	CM119593	TCC-TTC	Ser-Phe	Polycystic kidney disease 1
	CM124000	GCC-GAC	Ala-Asp	Polycystic kidney disease, autosomal

			dominant
CM022657	TGG-TGA	Trp-Term	Polycystic kidney disease 1
CM074415	ACC-ATC	Thr-Ile	Polycystic kidney disease 1
CM971178	TGG-TGA	Trp-Term	Polycystic kidney disease 1
CM124018	GGC-GCC	Gly-Ala	Polycystic kidney disease, autosomal dominant
CM076411	GGC-GTC	Gly-Val	Polycystic kidney disease 1
CM121781	GCC-GGC	Ala-Gly	Polycystic kidney disease 1
CM021122	ACC-ATC	Thr-Ile	Polycystic kidney disease 1
CM076425	TGG-TGA	Trp-Term	Polycystic kidney disease 1
CM1210916	TCC-TGC	Ser-Cys	Polycystic kidney disease 1
CM108788	GGC-GAC	Gly-Asp	Polycystic kidney disease 1
CM1210885	TGG-TGA	Trp-Term	Polycystic kidney disease 1
CM119592	TCC-TTC	Ser-Phe	Polycystic kidney disease 1
CM1210941	GGC-GAC	Gly-Asp	Polycystic kidney disease 1
CM124016	CCC-CTC	Pro-Leu	Polycystic kidney disease, autosomal dominant
CM121782	TGG-TGT	Trp-Cys	Polycystic kidney disease 1
CM124011	GGC-GAC	Gly-Asp	Polycystic kidney disease, autosomal dominant
CM010384	GCC-GAC	Ala-Asp	Polycystic kidney disease 1
CM020489	GGC-GAC	Gly-Asp	Polycystic kidney disease 1
CM076386	CCC-CTC	Pro-Leu	Polycystic kidney disease 1
CM1210944	ACC-ATC	Thr-Ile	Polycystic kidney disease 1
CM076381	ACC-ATC	Thr-Ile	Polycystic kidney disease 1
CM1210945	GGC-CGC	Gly-Arg	Polycystic kidney disease 1
CM121783	GGT-GAT	Gly-Asp	Polycystic kidney disease 1
CM1210923	GGG-GAG	Gly-Glu	Polycystic kidney disease 1
CM1210946	GGG-TGG	Gly-Trp	Polycystic kidney disease 1
CM010389	TGG-TGA	Trp-Term	Polycystic kidney disease 1

	CM1210898	TGG-TGA	Trp-Term	Polycystic kidney disease 1
	CM1210925	CCT-CTT	Pro-Leu	Polycystic kidney disease 1
	CM076426	TGG-TGA	Trp-Term	Polycystic kidney disease 1
	CM123944	TGG-TGA	Trp-Term	Polycystic kidney disease, autosomal dominant
	CM074436	GGC-CGC	Gly-Arg	Polycystic kidney disease 1
	CM076427	TGG-TGA	Trp-Term	Polycystic kidney disease 1
	CM057349	TCC-TTC	Ser-Phe	Polycystic kidney disease 1
	CM053384	TGG-TGA	Trp-Term	Polycystic kidney disease 1
	CM021656	CCC-CTC	Pro-Leu	Polycystic kidney disease 1
	CM1210948	GGC-GAC	Gly-Asp	Polycystic kidney disease 1
	CM1210949	GCC-GAC	Ala-Asp	Polycystic kidney disease 1
	CM090557	TGG-TCG	Trp-Ser	Polycystic kidney disease 1
	CM109266	CGG-CAG	Arg-Gln	Polycystic kidney disease 1
	CM124002	CGG-CTG	Arg-Leu	Polycystic kidney disease, autosomal dominant
	CM034563	TGG-TGA	Trp-Term	Polycystic kidney disease 1
	CM076428	TGG-TGA	Trp-Term	Polycystic kidney disease 1
	CM971194	TGG-TGA	Trp-Term	Polycystic kidney disease 1
	CM981546	GGC-GAC	Gly-Asp	Polycystic kidney disease 1
	CM992202	TGG-TGA	Trp-Term	Polycystic kidney disease 1
mutations create a PAM (26)	CM1210912	TGC-TGG	Cys-Trp	Polycystic kidney disease 1
	CM108786	GAT-GGT	Asp-Gly	Polycystic kidney disease 1
	CM034112	TGC-GGC	Cys-Gly	Polycystic kidney disease 1
	CM123999	TAC-TCC	Tyr-Ser	Polycystic kidney disease, autosomal dominant
	CM124017	GTC-GCC	Val-Ala	Polycystic kidney disease, autosomal dominant
	CM1210932	GTA-GGA	Val-Gly	Polycystic kidney disease 1

	CM076398	CTG-CGG	Leu-Arg	Polycystic kidney disease 1
	CM108793	CTG-CGG	Leu-Arg	Polycystic kidney disease 1
	CM1210913	GTG-GGG	Val-Gly	Polycystic kidney disease 1
	CM1210936	GTG-GGG	Val-Gly	Polycystic kidney disease 1
	CM034115	ACT-CCT	Thr-Pro	Polycystic kidney disease 1
	CM076383	CAC-CCC	His-Pro	Polycystic kidney disease 1
	CM074423	CTC-CCC	Leu-Pro	Polycystic kidney disease 1
	CM124020	TCG-TGG	Ser-Trp	Polycystic kidney disease, autosomal dominant ?
	CM108795	ATC-ACC	Ile-Thr	Polycystic kidney disease 1
	CM971180	CGC-CCC	Arg-Pro	Polycystic kidney disease 1, association with
	CM1210918	CGC-CCC	Arg-Pro	Polycystic kidney disease 1
	CM971184	ATC-ACC	Ile-Thr	Polycystic kidney disease 1
	CM074437	TTC-TCC	Phe-Ser	Polycystic kidney disease 1
	CM108796	AGC-AGG	Ser-Arg	Polycystic kidney disease 1
	CM002089	CAT-CCT	His-Pro	Polycystic kidney disease 1
	CM121784	TAC-TCC	Tyr-Ser	Polycystic kidney disease 1
	CM076399	CTG-CGG	Leu-Arg	Polycystic kidney disease 1
	CM076400	CTG-CGG	Leu-Arg	Polycystic kidney disease 1
	CM076394	CTC-CCC	Leu-Pro	Polycystic kidney disease 1
	CM993191	CAG-CCG	Gln-Pro	Polycystic kidney disease 1
**Gene name:NF2**				
mutations eliminate a PAM (4)	CM002815	TGG-TGA	Trp-Term	Neurofibromatosis 2
	CM070211	TGG-TGA	Trp-Term	Neurofibromatosis 2
	CM961033	GGC-TGC	Gly-Cys	Neurofibromatosis 2
	CM961034	TGG-TGA	Trp-Term	Neurofibromatosis 2
mutations create a PAM (4)	CM070212	TTG-TGG	Leu-Trp	Neurofibromatosis 2
	CM045421	AGC-ACC	Ser-Thr	Neurofibromatosis 2

	CM961040	CAG-CCG	Gln-Pro	Neurofibromatosis 2
	CM941099	GAA-GGA	Glu-Gly	Neurofibromatosis 2
**Gene name:COCH**				
mutations eliminate a PAM (4)	CM990378	CCA-TCA	Pro-Ser	Deafness, non-syndromic, autosomal dominant
	CM062521	GGG-TGG	Gly-Trp	Deafness, non-syndromic, autosomal dominant
	CM980383	GGA-GAA	Gly-Glu	Deafness, non-syndromic, autosomal dominant
	CM123274	CCT-CAT	Pro-His	Hearing loss, unilateral
mutations eliminate a PAM (3)	CM980382	GTA-GGA	Val-Gly	Deafness, non-syndromic, autosomal dominant
	CM076118	ATC-ACC	Ile-Thr	Deafness, non-syndromic, autosomal dominant
	CM108268	TTC-TCC	Phe-Ser	Deafness, non-syndromic, autosomal dominant
**Gene name:GJB2**				
mutations eliminate a PAM (37)	CM077935	TGG-TGA	Trp-Term	Hearing impairment, nonsyndromic
	CM034038	GGC-GAC	Gly-Asp	Deafness
	CM102365	GGG-GAG	Gly-Glu	Keratitis-ichthyosis-deafness syndrome
	CM020941	GGT-CGT	Gly-Arg	Keratitis-ichthyosis-deafness syndrome
	CM022364	GGT-GAT	Gly-Asp	Deafness
	CM000167	GGT-GTT	Gly-Val	Deafness, autosomal recessive 1
	CM042706	GGT-TGT	Gly-Cys	Deafness
	CM041348	TCC-TAC	Ser-Tyr	Deafness
	CM020942	TCC-TTC	Ser-Phe	Keratitis-ichthyosis-deafness syndrome
	CM085449	ACC-ATC	Thr-Ile	Deafness
	CM042708	TGG-TCG	Trp-Ser	Deafness, autosomal dominant 3
	CM993705	TGG-TGA	Trp-Term	Deafness, autosomal recessive 1
	CM980923	TGG-TGC	Trp-Cys	Deafness, autosomal dominant 3
	CM990689	GGA-GAA	Gly-Glu	Deafness, autosomal recessive 1
	CM030878	GCC-GTC	Ala-Val	Deafness ?
	CM050056	ACC-AAC	Thr-Asn	Deafness, autosomal recessive 1

	CM087021	CCA-CGA	Pro-Arg	Deafness, autosomal recessive 1
	CM073094	CCA-GCA	Pro-Ala	Deafness
	CM050057	GGC-CGC	Gly-Arg	Knuckle pads, hyperkeratosis and deafness
	CM000017	GGC-GCC	Gly-Ala	Deafness and palmoplantar keratoderma
	CM041350	GGC-GTC	Gly-Val	Deafness
	CM077556	CCC-CTC	Pro-Leu	Sensorineural hearing loss
	CM970681	TGG-TGA	Trp-Term	Deafness, autosomal recessive 1
	CM094088	GGG-GTG	Gly-Val	Deafness, autosomal recessive 1
	CM003935	ACC-AAC	Thr-Asn	Deafness
	CM077565	GGC-GAC	Gly-Asp	Sensorineural hearing loss
	CM034043	GGC-GCC	Gly-Ala	Deafness
	CM052247	GGC-GTC	Gly-Val	Vohwinkel syndrome
	CM051517	TGG-TGA	Trp-Term	Deafness, autosomal recessive 1
	CM101467	TGG-TGC	Trp-Cys	Deafness, nonsyndromic sensorineural
	CM000170	CCT-CGT	Pro-Arg	Deafness, autosomal recessive 1
	CM055280	CCT-TCT	Pro-Ser	Deafness, autosomal recessive 1
	CM085452	TCC-TTC	Ser-Phe	Focal palmoplantar keratoderma
	CM992895	CGG-CCG	Arg-Pro	Deafness, autosomal recessive 1
	CM042712	GGA-TGA	Gly-Term	Deafness
	CM077567	GTT-GGT	Val-Gly	Sensorineural hearing loss
	CM077557	CGG-CTG	Arg-Leu	Sensorineural hearing loss
mutations create a PAM (17)	CM102620	CAG-CCG	Gln-Pro	Sensorineural hearing loss
	CM000168	AGC-ACC	Ser-Thr	Deafness, autosomal recessive 1
	CM077555	ATG-AGG	Met-Arg	Sensorineural hearing loss
	CM065234	CTC-CCC	Leu-Pro	Deafness
	CM051511	GCA-GGA	Ala-Gly	Deafness, autosomal recessive 1
	CM084866	GAC-GCC	Asp-Ala	Keratitis-ichthyosis-deafness syndrome
	CM030879	ATC-ACC	Ile-Thr	Deafness
	CM022366	CAG-CCG	Gln-Pro	Deafness

	CM099689	GCG-CCG	Ala-Pro	Deafness
	CM091845	GCG-GGG	Ala-Gly	Sensorineural hearing loss
	CM053904	CAT-CCT	His-Pro	Deafness
	CM980928	AGT-AGG	Ser-Arg	Deafness, autosomal recessive 1
	CM013722	GAA-GGA	Glu-Gly	Deafness
	CM053902	ATG-AGG	Met-Arg	Deafness
	CM096519	GTC-GCC	Val-Ala	Hearing impairment, nonsyndromic
	CM012138	ATC-ACC	Ile-Thr	Deafness
	CM053903	ACT-CCT	Thr-Pro	Deafness
**Gene name:MYO7A**				
mutations eliminate a PAM (19)	CM004267	CGG-CCG	Arg-Pro	Usher syndrome 1b
	CM071875	CCC-CTC	Pro-Leu	Usher syndrome 1
	CM119666	ACC-ATC	Thr-Ile	Leber congenital amaurosis
	CM971013	GCC-GAC	Ala-Asp	Usher syndrome 1b
	CM004270	GCC-GTC	Ala-Val	Usher syndrome 1b
	CM961014	CCC-CTC	Pro-Leu	Usher syndrome 1b
	CM004271	GGC-GAC	Gly-Asp	Usher syndrome 1b
	CM041044	GGC-CGC	Gly-Arg	Deafness, non-syndromic, autosomal dominant
	CM114224	GGC-GTC	Gly-Val	Usher syndrome 1
	CM061141	CGG-CCG	Arg-Pro	Usher syndrome 1
	CM061139	CCC-CGC	Pro-Arg	Usher syndrome 1
	CM990902	AGG-AGC	Arg-Ser	Usher syndrome 1b
	CM1110175	TGG-TGA	Trp-Term	Usher syndrome 1
	CM071879	GCC-GTC	Ala-Val	Usher syndrome 1
	CM091798	GCC-GAC	Ala-Asp	Usher syndrome 1b
	CM117301	GGA-TGA	Gly-Term	Usher syndrome 1b
	CM091799	GGA-GAA	Gly-Glu	Usher syndrome 1b
	CM971018	GGG-GAG	Gly-Glu	Usher syndrome 1b
	CM004275	GGC-GAC	Gly-Asp	Usher syndrome 1b
mutations create a PAM (5)	CM1110176	CGT-CCT	Arg-Pro	Usher syndrome 1
	CM071872	CGT-GGT	Arg-Gly	Usher syndrome 1
	CM971012	CGC-CCC	Arg-Pro	Deafness, non-syndromic, autosomal recessive
	CM061138	CAG-CCG	Gln-Pro	Usher syndrome 1
	CM071874	ATC-ACC	Ile-Thr	Usher syndrome 1
**Gene name:TECTA**				

mutations eliminate a PAM (3)	CM115247	GCC-GTC	Ala-Val	Deafness, autosomal dominant
	CM115251	CCC-CGC	Pro-Arg	Deafness, autosomal dominant
	CM981906	GGT-GAT	Gly-Asp	Deafness, autosomal dominant 12
mutations create a PAM (3)	CM014985	TGC-GGC	Cys-Gly	Deafness
	CM045200	TGC-GGC	Cys-Gly	Deafness, autosomal dominant 12
	CM992436	TGC-TCC	Cys-Ser	Deafness, autosomal dominant 12
**Gene name:DSPP**				
mutations eliminate a PAM (3)	CM040731	GCC-GTC	Ala-Val	Dentinogenesis imperfecta type II
	CM010216	CCA-ACA	Pro-Thr	Dentinogenesis imperfecta type I
	CM074146	CCA-TCA	Pro-Ser	Dentinogenesis imperfecta type II
**Gene name:WFS1**				
mutations eliminate a PAM (23)	CM087006	GGG-GAG	Gly-Glu	Wolfram syndrome
	CM043876	TGG-TGA	Trp-Term	Wolfram syndrome
	CM112215	ACC-ATC	Thr-Ile	Wolfram syndrome
	CM050354	TGG-TGA	Trp-Term	Wolfram syndrome
	CM992984	GGC-CGC	Gly-Arg	Wolfram syndrome
	CM043877	ACC-ATC	Thr-Ile	Wolfram syndrome
	CM050355	AGG-AGT	Arg-Ser	Wolfram syndrome
	CM087001	ACC-AGC	Thr-Ser	Wolfram syndrome
	CM033825	CCC-CGC	Pro-Arg	Wolfram syndrome
	CM032679	TGG-TGA	Trp-Term	Wolfram syndrome
	CM982041	TGG-TGA	Trp-Term	Wolfram syndrome
	CM020990	GGG-GAG	Gly-Glu	Sensorineural hearing loss
	CM020991	GGG-GTG	Gly-Val	Sensorineural hearing loss
	CM073425	TGG-TTG	Trp-Leu	Sensorineural hearing loss
	CM982042	GGC-GTC	Gly-Val	Wolfram syndrome
	CM992989	TGG-TGT	Trp-Cys	Wolfram syndrome
	CM090455	GGC-GAC	Gly-Ser	Wolfram syndrome
	CM112220	CCG-TCG	Pro-Ser	Wolfram syndrome
	CM041102	GGC-CGC	Gly-Arg	Wolfram syndrome
	CM012813	GGC-CGC	Gly-Arg	Wolfram syndrome

	CM013446	GGC-GAC	Gly-Asp	Sensorineural hearing loss
	CM052943	CGG-CCG	Arg-Pro	Sensorineural hearing loss
	CM053438	TGG-TGA	Trp-Term	Wolfram syndrome
mutations create a PAM (15)	CM083208	CGC-CCC	Arg-Pro	Wolfram syndrome
	CM015259	ATG-AGG	Met-Arg	Wolframin variant
	CM112217	TCG-TGG	Ser-Trp	Wolfram syndrome
	CM118453	AGC-AGG	Ser-Arg	Wolfram syndrome
	CM043878	CTG-CGG	Leu-Arg	Wolfram syndrome
	CM024439	GAG-GGG	Glu-Gly	Wolframin variant
	CM090453	CTG-CGG	Leu-Arg	Wolfram syndrome
	CM119609	ATG-AGG	Met-Arg	Wolfram syndrome
	CM108446	GCG-GGG	Ala-Gly	Wolfram syndrome
	CM081852	CGC-CCC	Arg-Pro	Sensorineural hearing loss
	CM087004	TGC-GGC	Cys-Gly	Wolfram syndrome
	CM112219	CTC-CCC	Leu-Pro	Wolfram syndrome
	CM015267	GAC-GGC	Asp-Gly	Wolframin variant
	CM118452	GTG-GGG	Val-Gly	Wolfram syndrome
	CM031404	CGC-CCC	Arg-Pro	Wolfram syndrome
**Gene name:F11**				
mutations eliminate a PAM (27)	CM091620	GGT-CGT	Gly-Arg	Factor XI deficiency
	CM062620	CCA-CAA	Pro-Gln	Factor XI deficiency
	CM097438	CCC-CTC	Pro-Leu	Factor XI deficiency
	CM056952	CCA-ACA	Pro-Thr	Factor XI deficiency
	CM086812	GGG-GCG	Gly-Ala	Factor XI deficiency
	CM030841	GGC-GAC	Gly-Asp	Factor XI deficiency
	CM030842	GGG-GAG	Gly-Glu	Factor XI deficiency
	CM066064	CCT-TCT	Pro-Ser	Factor XI deficiency
	CM053239	TCC-TTC	Ser-Phe	Factor XI deficiency
	CM990520	TGG-TGC	Trp-Cys	Factor XI deficiency
	CM051915	GGA-GAA	Gly-Glu	Factor XI deficiency
	CM121069	GGT-GAT	Gly-Asp	Factor XI deficiency
	CM950373	ACC-ATC	Thr-Ile	Factor XI deficiency
	CM080237	ACC-ATC	Thr-Ile	Factor XI deficiency
	CM920987	GGA-GAA	Gly-Glu	Factor XI deficiency
	CM042342	GGA-GCA	Gly-Ala	Factor XI deficiency
	CM091842	GGA-GCA	Gly-Ala	Factor XI deficiency
	CM090244	TGG-TTG	Trp-Leu	Factor XI deficiency
	CM970459	ACC-AAC	Thr-Asn	Factor XI deficiency
	CM041757	GGC-GTC	Gly-Val	Factor XI deficiency

	CM082626	TGG-TGC	Trp-Cys	Factor XI deficiency
	CM056953	TGG-TGT	Trp-Cys	Factor XI deficiency
	CM044883	TGG-TGC	Trp-Cys	Factor XI deficiency
	CM051916	CCC-CTC	Pro-Leu	Factor XI deficiency
	CM043486	GGA-GAA	Gly-Glu	Factor XI deficiency
	CM041758	TGG-TCG	Trp-Ser	Factor XI deficiency
	CM082624	GGC-TGC	Gly-Cys	Factor XI deficiency
mutations create a PAM (16)	CM076174	GTC-GCC	Val-Ala	Factor XI deficiency
	CM097406	AGC-AGG	Ser-Arg	Factor XI deficiency
	CM116684	CAG-CCG	Gln-Pro	Factor XI deficiency
	CM080239	ACT-CCT	Thr-Pro	Factor XI deficiency
	CM095841	TGT-TGG	Cys-Trp	Factor XI deficiency
	CM125455	ACT-CCT	Thr-Pro	Factor XI deficiency
	CM095842	CGA-CCA	Arg-Pro	Factor XI deficiency
	CM056954	TGC-GGC	Cys-Gly	Factor XI deficiency
	CM035504	TAC-TCC	Tyr-Ser	Factor XI deficiency
	CM044881	CTG-CCG	Leu-Pro	Factor XI deficiency
	CM051039	TAC-TCC	Tyr-Ser	Factor XI deficiency
	CM082621	CAC-CCC	His-Pro	Factor XI deficiency
	CM035501	ACA-CCA	Thr-Pro	Factor XI deficiency
	CM062630	TGC-TGG	Cys-Trp	Factor XI deficiency
	CM119142	GAT-GGT	Asp-Gly	Factor XI deficiency
	CM097457	GAT-GGT	Asp-Gly	Factor XI deficiency
**Gene name:HFE**				
mutations eliminate a PAM (4)	CM032270	AGG-AGC	Arg-Ser	Haemochromatosis
	CM990719	GGG-CGG	Gly-Arg	Haemochromatosis
	CM015326	GCC-GTC	Ala-Val	Haemochromatosis
	CM990722	AGG-ATG	Arg-Met	Haemochromatosis
mutations create a PAM (3)	CM091838	TTG-TGG	Leu-Trp	Haemochromatosis
	CM004391	TGC-TCC	Cys-Ser	Haemochromatosis
	CM032271	CAG-CCG	Gln-Pro	Haemochromatosis
**Gene name:F8**				
mutations eliminate a PAM (20)	CM0911148	GGT-GAT	Gly-Asp	Haemophilia A
	CM010836	GGT-TGT	Gly-Cys	Haemophilia A
	CM123839	TGG-TGA	Trp-Term	Haemophilia A
	CM121364	GGT-CGT	Gly-Arg	Haemophilia A
	CM960537	GGT-GAT	Gly-Asp	Haemophilia A

	CM106046	GGT-GCT	Gly-Ala	Haemophilia A
	CM129821	GGT-CGT	Gly-Arg	Haemophilia A
	CM080305	GGT-GAT	Gly-Asp	Haemophilia A
	CM014182	GGT-GCT	Gly-Ala	Haemophilia A
	CM920250	GGT-GTT	Gly-Val	Haemophilia A
	CM0911146	GGT-TGT	Gly-Cys	Haemophilia A
	CM080330	CCT-TCT	Pro-Ser	Haemophilia A
	CM062683	TCC-TTC	Ser-Phe	Haemophilia A
	CM055234	GGT-CGT	Gly-Arg	Haemophilia A
	CM073047	GGT-GCT	Gly-Ala	Haemophilia A
	CM054673	GGT-GTT	Gly-Val	Haemophilia A
	CM082663	GGT-TGT	Gly-Cys	Haemophilia A
	CM080328	TCC-TGC	Ser-Cys	Haemophilia A
	CM081247	CCA-CGA	Pro-Arg	Haemophilia A
	CM930210	GGA-CGA	Gly-Arg	Haemophilia A
mutations create a PAM (12)	CM025245	ATG-AGG	Met-Arg	Haemophilia A
	CM990526	AGT-AGG	Ser-Arg	Haemophilia A
	CM960535	CTG-CGG	Leu-Arg	Haemophilia A
	CM053258	GTG-GGG	Val-Gly	Haemophilia A
	CM053253	ACT-CCT	Thr-Pro	Haemophilia A
	CM071725	ATC-ACC	Ile-Thr	Haemophilia A
	CM025251	GCT-CCT	Ala-Pro	Haemophilia A
	CM980665	GAT-GGT	Asp-Gly	Haemophilia A
	CM102433	GTC-GCC	Val-Ala	Haemophilia A
	CM087473	AAC-ACC	Asn-Thr	Haemophilia A
	CM080311	GTC-GGC	Val-Gly	Haemophilia A
	CM082633	AGT-AGG	Ser-Arg	Haemophilia A
**Gene name:F9**				
mutations eliminate a PAM (22)	CM960581	GGA-GTA	Gly-Val	Haemophilia B
	CM045755	GGA-TGA	Gly-Term	Haemophilia B
	CM057685	GCC-GAC	Ala-Asp	Haemophilia B
	CM970482	GCC-GTC	Ala-Val	Warfarin sensitivity
	CM940414	CGG-CTG	Arg-Leu	Haemophilia B
	CM980721	AGG-ACG	Arg-Thr	Haemophilia B
	CM940411	AGG-AGC	Arg-Ser	Haemophilia B
	CM995237	AGG-AGT	Arg-Ser	Haemophilia B
	CM960568	GGT-GAT	Gly-Asp	Haemophilia B
	CM940418	GGG-GCG	Gly-Ala	Haemophilia B
	CM053885	GGG-GTG	Gly-Val	Haemophilia B
	CM940449	GGA-GTA	Gly-Val	Haemophilia B
	CM010262	CCA-ACA	Pro-Thr	Haemophilia B

	CM940456	CCA-CAA	Pro-Gln	Haemophilia B
	CM990569	CCA-CGA	Pro-Arg	Haemophilia B
	CM940458	CCA-GCA	Pro-Ala	Haemophilia B
	CM940459	CCA-TCA	Pro-Ser	Haemophilia B
	CM000151	GGC-GAC	Gly-Asp	Haemophilia B
	CM940463	GGC-GTC	Gly-Val	Haemophilia B
	CM940467	GGC-CGC	Gly-Arg	Haemophilia B
	CM940465	GGC-GAC	Gly-Asp	Haemophilia B
	CM960576	GGC-TGC	Gly-Cys	Haemophilia B
mutations create a PAM (16)	CM001676	TGT-GGT	Cys-Gly	Haemophilia B
	CM940505	TGT-TGG	Cys-Trp	Haemophilia B
	CM045742	AAC-ACC	Asn-Thr	Haemophilia B
	CM990567	TCA-CCA	Ser-Pro	Haemophilia B
	CM071738	GAA-GGA	Glu-Gly	Haemophilia B
	CM045770	GAA-GGA	Glu-Gly	Haemophilia B
	CM098269	TGT-GGT	Cys-Gly	Haemophilia B
	CM001669	GAA-GGA	Glu-Gly	Haemophilia B
	CM950426	CGA-CCA	Arg-Pro	Haemophilia B
	CM045771	GAA-GGA	Glu-Gly	Haemophilia B
	CM940446	GAT-GGT	Asp-Gly	Haemophilia B
	CM950427	GAT-GGT	Asp-Gly	Haemophilia B
	CM940451	CAG-CCG	Gln-Pro	Haemophilia B
	CM940454	TGT-TGG	Cys-Trp	Haemophilia B
	CM990570	TGC-TCC	Cys-Ser	Haemophilia B
	CM057696	TGC-TGG	Cys-Trp	Haemophilia B
**Gene name:G6PD**				
mutations eliminate a PAM (28)	CM950496	GCC-GGC	Ala-Gly	Glucose-6-phosphate dehydrogenase deficiency
	CM952134	TGG-TGC	Trp-Cys	Glucose-6-phosphate dehydrogenase deficiency
	CM026005	CCC-CAC	Pro-His	Glucose-6-phosphate dehydrogenase deficiency
	CM870009	CCC-CTC	Pro-Leu	Glucose-6-phosphate dehydrogenase deficiency
	CM061777	CCA-TCA	Pro-Ser	Glucose-6-phosphate dehydrogenase deficiency
	CM920277	TCC-TGC	Ser-Cys	Glucose-6-phosphate dehydrogenase deficiency
	CM930268	GGG-GTG	Gly-Val	Glucose-6-phosphate dehydrogenase

			deficiency
CM930269	GGC-GAC	Gly-Asp	Glucose-6-phosphate dehydrogenase deficiency
CM880032	TCC-TTC	Ser-Phe	Glucose-6-phosphate dehydrogenase deficiency
CM890051	CGG-CTG	Arg-Leu	Glucose-6-phosphate dehydrogenase deficiency
CM100166	CCC-CTC	Pro-Leu	Glucose-6-phosphate dehydrogenase deficiency
CM950503	TCC-TTC	Ser-Phe	Glucose-6-phosphate dehydrogenase deficiency
CM973143	GGG-GAG	Gly-Glu	Glucose-6-phosphate dehydrogenase deficiency
CM973144	GGG-GAG	Gly-Glu	Glucose-6-phosphate dehydrogenase deficiency
CM980789	CCC-CAC	Pro-His	Glucose-6-phosphate dehydrogenase deficiency
CM950506	GCC-GAC	Ala-Asp	Glucose-6-phosphate dehydrogenase deficiency
CM942061	GGG-GTG	Gly-Val	Glucose-6-phosphate dehydrogenase deficiency
CM973147	GCC-GTC	Ala-Val	Glucose-6-phosphate dehydrogenase deficiency
CM940798	CCC-CTC	Pro-Leu	Glucose-6-phosphate dehydrogenase deficiency
CM056962	ACC-AAC	Thr-Asn	Glucose-6-phosphate dehydrogenase deficiency
CM044626	CCG-TCG	Pro-Ser	Glucose-6-phosphate dehydrogenase deficiency
CM920287	GGC-GAC	Gly-Asp	Glucose-6-phosphate dehydrogenase deficiency
CM930280	GGC-TGC	Gly-Cys	Glucose-6-phosphate dehydrogenase deficiency
CM102665	GGG-GTG	Gly-Val	Glucose-6-phosphate dehydrogenase deficiency
CM970550	CCA-CGA	Pro-Arg	Glucose-6-phosphate dehydrogenase deficiency

	CM056326	CCC-CGC	Pro-Arg	Glucose-6-phosphate dehydrogenase deficiency
	CM930285	GGC-GTC	Gly-Val	Glucose-6-phosphate dehydrogenase deficiency
	CM973153	CCC-CTC	Pro-Leu	Glucose-6-phosphate dehydrogenase deficiency
mutations create a PAM (14)	CM930263	ATC-ACC	Ile-Thr	Glucose-6-phosphate dehydrogenase deficiency
	CM930264	CTC-CCC	Leu-Pro	Glucose-6-phosphate dehydrogenase deficiency
	CM950499	CTC-CCC	Leu-Pro	Glucose-6-phosphate dehydrogenase deficiency
	CM962574	AAC-ACC	Asn-Thr	Glucose-6-phosphate dehydrogenase deficiency
	CM920280	CGC-CCC	Arg-Pro	Glucose-6-phosphate dehydrogenase deficiency
	CM110926	GAC-GGC	Asp-Gly	Glucose-6-phosphate dehydrogenase deficiency
	CM077931	GTC-GCC	Val-Ala	Glucose-6-phosphate dehydrogenase deficiency
	CM051484	ATC-ACC	Ile-Thr	Glucose-6-phosphate dehydrogenase deficiency
	CM970548	TGC-TGG	Cys-Phe	Glucose-6-phosphate dehydrogenase deficiency
	CM077930	ATC-ACC	Ile-Thr	Glucose-6-phosphate dehydrogenase deficiency
	CM950512	CGC-GGC	Arg-Gly	Glucose-6-phosphate dehydrogenase deficiency
	CM970549	GTG-GGG	Val-Gly	Glucose-6-phosphate dehydrogenase deficiency
	CM920289	CGC-CCC	Arg-Pro	Glucose-6-phosphate dehydrogenase deficiency
	CM930284	CGT-CCT	Arg-Pro	Glucose-6-phosphate dehydrogenase deficiency
**Gene name:HMBS**				
mutations eliminate a	CM970727	GGT-AGT	Gly-Ser	Porphyria, acute intermittent

PAM (22)				
	CM104051	GGT-GAT	Gly-Asp	Porphyria, acute intermittent
	CM023941	TCC-TTC	Ser-Phe	Porphyria, acute intermittent
	CM930402	GGA-AGA	Gly-Arg	Porphyria, acute intermittent
	CM104055	GCC-GAC	Ala-Asp	Porphyria, acute intermittent
	CM940937	CGG-CAG	Arg-Gln	Porphyria, acute intermittent
	CM950639	CCT-CTT	Pro-Leu	Porphyria, acute intermittent
	CM900130	CGG-CAG	Arg-Gln	Porphyria, acute intermittent
	CM900131	CGG-CAG	Arg-Gln	Porphyria, acute intermittent
	CM910224	TGG-TAG	Trp-Term	Porphyria, acute intermittent
	CM970733	GGC-GAC	Gly-Asp	Porphyria, acute intermittent
	CM990738	GCC-GAC	Ala-Asp	Porphyria, acute intermittent
	CM083526	GGC-GAC	Gly-Asp	Porphyria, acute intermittent
	CM040225	GGT-AGT	Gly-Ser	Porphyria, acute intermittent
	CM930405	GCC-GTC	Ala-Val	Porphyria, acute intermittent
	CM022218	GGC-GAC	Gly-Asp	Porphyria, acute intermittent
	CM950646	GGA-AGA	Gly-Arg	Porphyria, acute intermittent
	CM0911052	GGA-GAA	Gly-Glu	Porphyria, acute intermittent
	CM940946	TGG-TAG	Trp-Term	Porphyria, acute intermittent
	CM940947	TGG-TGA	Trp-Term	Porphyria, acute intermittent
	CM992341	GGC-AGC	Gly-Ser	Porphyria, acute intermittent
	CM970741	GGC-GAC	Gly-Asp	Porphyria, acute intermittent
mutations create a PAM (28)	CM090525	CGC-CCC	Arg-Pro	Porphyria, acute intermittent
	CM992340	CAG-CCG	Gln-Lys	Porphyria, acute intermittent
	CM040224	CAG-CGG	Gln-Arg	Porphyria, acute intermittent
	CM004871	GAG-GGG	Glu-Gly	Porphyria, acute intermittent
	CM040753	CTT-CCT	Leu-Pro	Porphyria, acute intermittent
	CM990735	CTG-CGG	Leu-Arg	Porphyria, acute

				intermittent
	CM990736	GTG-GGG	Val-Gly	Porphyria, acute intermittent
	CM022214	CTG-CCG	Leu-Pro	Porphyria, acute intermittent
	CM950638	AAG-AGG	Lys-Arg	Porphyria, acute intermittent
	CM022215	GAC-GGC	Asp-Gly	Porphyria, acute intermittent
	CM022216	ATC-ACC	ILE-Thr	Porphyria, acute intermittent
	CM023943	GCT-GGT	Ala-Gly	Porphyria, acute intermittent
	CM083525	CGA-CCA	Arg-Pro	Porphyria, acute intermittent
	CM920345	CTG-CGG	Leu-Arg	Porphyria, acute intermittent
	CM061060	CAG-CGG	Gln-Arg	Porphyria, acute intermittent
	CM950642	CGA-GGA	Arg-Gly	Porphyria, acute intermittent
	CM0911048	CTG-CCG	Leu-Pro	Porphyria, acute intermittent
	CM062775	CTG-CCG	Leu-Pro	Porphyria, acute intermittent
	CM950644	CTG-CGG	Leu-Arg	Porphyria, acute intermittent
	CM040226	CTG-CCG	Leu-Pro	Porphyria, acute intermittent
	CM0911049	CTG-CGG	Leu-Arg	Porphyria, acute intermittent
	CM023945	CTG-CCG	Leu-Pro	Porphyria, acute intermittent
	CM002280	GCT-GGT	Ala-Gly	Porphyria, acute intermittent
	CM0911051	CTG-CGG	leu-Arg	Porphyria, acute intermittent
	CM980986	CTG-CCG	Leu-Pro	Porphyria, acute intermittent
	CM104059	CTG-CCG	Leu-Pro	Porphyria, acute intermittent
	CM104058	CTG-CGG	Leu-Arg	Porphyria, acute intermittent
	CM022219	CTG-CCG	Leu-Pro	Porphyria, acute intermittent
**Gene name:CFTR** mutations eliminate a PAM (41)	CM970256	CCT-CTT	Pro-Leu	Cystic fibrosis
	CM0910748	TCC-TAC	Ser-Tyr	Azoospermia
	CM962449	TCC-TTC	Ser-Phe	Cystic fibrosis
	CM983534	TGG-TGA	Trp-Term	Cystic fibrosis
	CM970257	TGG-TGT	Trp-Cys	Cystic fibrosis

CM043457	GGA-AGA	Gly-Arg	Cystic fibrosis
CM057570	GGA-CGA	Gly-Arg	Cystic fibrosis
CM940234	GGA-GAA	Gly-Glu	Cystic fibrosis
CM920138	GGA-TGA	Gly-Term	Cystic fibrosis
CM994782	TGG-TGA	Trp-Term	Cystic fibrosis
CM930097	CCT-CTT	Pro-Leu	Cystic fibrosis
CM076095	CCT-TCT	Pro-Ser	Cystic fibrosis
CM962450	GCC-GAC	Ala-Asp	Cystic fibrosis
CM003236	CGG-CAG	Arg-Gln	Cystic fibrosis
CM931141	TGG-TAG	Trp-Term	Cystic fibrosis
CM910068	GGA-GAA	Gly-Glu	Cystic fibrosis
CM970262	GGA-GTA	Gly-Val	Cystic fibrosis
CM920981	GGA-TGA	Gly-Term	Cystic fibrosis
CM950236	TCC-TTC	Ser-Phe	Cystic fibrosis
CM962452	CCG-GCG	Pro-Ala	Cystic fibrosis
CM940243	GGC-GAC	Gly-Asp	Cystic fibrosis
CM941967	CCA-TCA	Pro-Ser	Cystic fibrosis
CM970264	GCC-GAC	Ala-Asp	Cystic fibrosis
CM067763	GGA-GTA	Gly-Val	Cystic fibrosis
CM024670	GCC-GTC	Ala-Val	Hypertrypsinaemia, neonatal
CM970273	CGG-CTG	Arg-Leu	Congenital absence of vas deferens
CM024674	GCC-GAC	Ala-Asp	Congenital absence of vas deferens
CM980335	CCT-GCT	Pro-Ala	Congenital absence of vas deferens
CM970277	GGT-GTT	Gly-Val	Congenital absence of vas deferens
CM980337	GGT-GCT	Gly-Ala	Congenital absence of vas deferens
CM067770	CCT-TCT	Pro-Ser	Congenital absence of vas deferens
CM920164	GGA-GCA	Gly-Ala	Congenital absence of vas deferens
CM980348	AGG-ATG	Arg-Met	Congenital absence of vas deferens
CM004892	CCA-CGA	Pro-Arg	Congenital absence of vas deferens
CM972957	CCA-TCA	Pro-Ser	Congenital absence of vas deferens
CM015360	GGT-GCT	Gly-Ala	Congenital absence of vas deferens
CM057584	GGA-CGA	Gly-Arg	Cystic fibrosis with pancreatic insufficiency
CM113212	GCC-GTC	Ala-Val	Congenital absence of vas deferens
CM055122	CCA-TCA	Pro-Ser	Congenital absence of vas deferens
CM067755	GAA-GGA	Glu-Gly	Congenital absence

				of vas deferens
	CM024698	TCC-TTC	Ser-Phe	Obstructive azoospermia
mutations create a PAM (23)	CM010791	ATG-AGG	Met-Arg	Asthma
	CM067745	AGC-GGC	Ser-Gly	Cystic fibrosis
	CM920140	GAT-GGT	Asp-Gly	Cystic fibrosis
	CM952045	TCT-CCT	Ser-Pro	Cystic fibrosis
	CM003234	GAT-GGT	Asp-Gly	Cystic fibrosis
	CM057577	CTG-CCG	Leu-Pro	Cystic fibrosis
	CM126469	CGA-GGA	Arg-Gly	Cystic fibrosis
	CM015351	CAG-CCG	Gln-Pro	Cystic fibrosis
	CM057578	CTG-CGG	Leu-Arg	Cystic fibrosis
	CM074735	GCT-GGT	Ala-Gly	Cystic fibrosis
	CM930100	CGC-CCC	Arg-Pro	Cystic fibrosis
	CM005456	CGC-GGC	Arg-Gly	Cystic fibrosis
	CM051410	CTC-CCC	Leu-Pro	Cystic fibrosis
	CM970269	ATG-AGG	Met-Arg	Congenital absence of vas deferens
	CM980336	GAT-GGT	Asp-Gly	Congenital absence of vas deferens
	CM990361	GAA-GGA	Glu-Gly	Hypertrypsinaemia, low sweat chloride
	CM983666	GAT-GGT	Asp-Gly	Congenital absence of vas deferens
	CM077237	AGC-AGG	Ser-Arg	Congenital absence of vas deferens
	CM980347	CGA-GGA	Arg-Gly	Congenital absence of vas deferens
	CM970290	GTG-GGG	Val-Gly	Congenital absence of vas deferens
	CM074734	GAC-GGC	Asp-Gly	Respiratory symptoms of cystic fibrosis
	CM015359	GCT-CCT	Ala-Pro	Congenital absence of vas deferens
	CM941983	GAT-GGT	Asp-Gly	Congenital absence of vas deferens
**Gene name:UROS** mutations eliminate a PAM (5)	CM900224	CCT-CTT	Pro-Leu	Porphyria, erythropoietic
	CM128678	GGA-AGA	Gly-Arg	Porphyria, erythropoietic
	CM951266	GGC-AGC	Gly-Ser	Porphyria, erythropoietic
	CM067488	GGC-GTC	Gly-Val	Porphyria, erythropoietic
	CM961413	CCA-CAA	Pro-Gln	Porphyria, erythropoietic

mutations create a PAM (4)	CM042498	TCT-CCT	Ser-Pro	Porphyria, erythropoietic
	CM961411	CAG-CCG	Gln-Pro	Porphyria, erythropoietic
	CM961412	TCT-CCT	Ser-Pro	Porphyria, erythropoietic
	CM067487	CTT-CCT	Leu-Pro	Porphyria, erythropoietic

Supplemental Table 6-1.DNA sequencing results of human iPSC transfected with RNP targeting PDCD1 gene. Mutated sites are in red.

WT	ACGCCCGTTGGGCAGTTGTGTGACACGGAAGCGGCAGTCCTGGC
**Experiment Batch 1**	
Clone#1.	ACGCCCGTTGGGCAGTTGTGTGACACGGAAGCGGCAGTCCTGGC
Clone#2.	ACGCCCGTTGGGCAGTTGTG-----------CGGCAGTCCTGGC
Clone#3.	ACGCCCGTTGGGCAGTTGTGTGACACGGAAGCGGCAGTCCTGGC
Clone#4.	ACGCCCGTTG----------------------GGCAGTCCTGG
Clone#5.	ACGCCCGTTG----------------------GGCAGTCCTGGC
Clone#6.	ACGCCCGTTGGGCAGTTGTGTGACACGGAAGCGGCAGTCCTGGC
Clone#7.	ACGCC-----------------------AAGCGGCAGTCCTGGC
Clone#8.	ACGCCCGTTGGGCAGTTGTGTGACA------CGGCAGTCCTGGC
Clone#9.	ACGCCCGTTGGGCAGTTGTGTGAC----AAGCGGCAGTCCTGGC
Clone#10.	ACGCCCGTTGGGCAGTTGTGTGACACGGAAGCGGCAGTCCTGGC
Clone#10.	ACGCCCGTTGGGCAGTTGTGTGACACGGAAGCGGCAGTCCTGGC
**Experiment Batch 2**	
Clone#12.	ACGCCCGTTGGGCAGTTGTGTGACACGGAAGCGGCAGTCCTGGC
Clone#13.	ACGCCCGTTGGGCAGTTGTGTGACACGGAAGCGGCAGTCCTGGC
Clone#14.	ACGCCCGTTGGGCAGTTGTGTGACACGGAAGCGGCAGTCCTGGC
Clone#15.	ACGCCCGTTGGGCAGTTGTGTGACACGGAAGCGGCAGTCCTGGC
Clone#16.	ACGCCCGTTGGGCAGTTGTGTGACA------CGGCAGTCCTGGC
Clone#17.	ACGCCCGTTGGGCAGTTGT---------AAGCGGCAGTCCTGGC
Clone#18.	ACGCCCGTTGGGCAGTTGTGTGACA------CGGCAGTCCTGGC
Clone#19.	ACGCCCGTTGGGCAGTTG----------------CAGTCCTGGC
Clone#20.	ACGCCCGTTGGGCAGTTGTGTGACACGGAAGCGGCAGTCCTGGC
Clone#21.	ACGCCCGTTGG----------------------GCAGTCCTGGC
Clone#22.	ACGCCCGTTGGGCAGTTGTGTGACACGGAAGCGGCAGTCCTGGC
Clone#23.	ACGCCCGTTGGGCAGTTGTGTGACACGGAAGCGGCAGTCCTGGC

Total clones sequenced 23	
Wildtype	Indels
52.2%	47.8%

Supplemental Table 6-2.DNA sequencing results of human iPSC transfected with RNP targeting PDCD1 gene and ssODN template carrying G deletion in PAM. Mutated sites are in red.

WT	ACGCCCGTTGGGCAG-TT---GTGTGAC-AC-G-GAAGCGGCAGTCCTGGC
Experiment Batch 1	
Clone#1.	ACGCCCGTTGGGCAG-TT---GTGTGAC-AC-G-GAAGCGGCAGTCCTGGC
Clone#2.	ACGCCCGTTGGGCAG-TT---GTGTGAC-AC---GAAGCGGCAGTCCTGGC
Clone#3.	ACGCCCGTTGGGCAG-TT---GTGTGAC-AC-G-GAAGC-GCAGTCCTGGC
Clone#4.	ACGCCCGTTGGGCAG-TT---GTGTGAC-AC-G-GAAGCGGCAGTCCTGGC
Clone#5.	ACGCCCGTTGGGCAG-TT---GTGTGAC-AC----AAGCGGCAGTCCTGGC
Clone#6.	ACGCCCGTTGG---G-TT----------------AAGCGGCAGTCCTGGC
Clone#7.	ACGCCCGTTGGGCAG-TT---GTGTGAC-AC-G-GAAGCGGCAGTCCTGGC
Clone#8.	ACGCCCGTTGGGCAG-TT---GTGTGAC-AC-G-GAAGCGGCAGTCCTGGC
Clone#9.	ACGCCCGTTGGGCAG-TT---GTGTGAC-AC-G-GAAGCGGCAGTCCTGGC
Clone#10.	--------------------------------------GGCAGTCCTGGC
Clone#11.	ACGCCCGTTG----------------------------GGCAGTCCTGGC
Clone#12.	ACGCCCGTTGGGCAG-TT---GTGTGA--------AAGCGGCAGTCCTGGC
Clone#13.	ACGCCCGTTGGGCAG-TT---GTGTGAC-AC-G-GAAGC-GCAGTCCTGGC
Clone#14.	ACGCCCGTCGGGCAGTTT---GTGTGAC-AC-G-GAAGCGGCAGTCCTGGC
Clone#15.	ACGCCCGTTGGGCAG-TT---GTGTGAC-AC---GAAGCGGCAGTCCTGGC
Clone#16.	ACGCCCGTTGGGCAG-TT---GTGTGAC-AC-G-GAAGCGGCAGTCCTGGC
Clone#17.	ACGCCCGTTGGGCAG-TT---GTGTGAC-AC-G-GAAGCGGCAGTCCTGGC
Clone#18.	ACGCCCGTTGGGCAG-TT---GTGTGAC-AC-G-GAAGC-GCAGTCCTGGC
Clone#19.	ACGCCCGTTGGGCAG-TT---GTGTGAC-AC-G-GAAGC-GCAGTCCTGGC
Clone#20.	ACGCCCGTTGGGCAG-TT---GTGTGAC-AC-G-GAAGCGGCAGTCCTGGC
Clone#21.	ACGCCCGTTGGGCAG-TT---GTGTGAC-AC------GCGGCAGTCCTGGC
Clone#22.	ACGCCCGTTGGGCAG-TT---GTGTGAC-AC-G-GAAGC-GCAGTCCTGGC
Clone#23.	ACGCCCGTTGGGCAG-TT---GTGTGAC-AC-G-GAAGC-GCAGTCCTGGC
Experiment Batch 2	
Clone#24.	ACGCCCGTTGGGCAG-TT---GTGT-------G-GAAGCGGCAGTCCTGGC

Clone#25. ACGCCCGTTGGGCAG-TT---GTGTGAC-AC-G-GAAGCGGCAGTCCTGGC

Clone#26. ACGCCCGTTGGGCAG-TT---GTGTGACG-C—G--AAGCGGCAGTCCTGGC

Clone#27. ACGCCCGTTGGGCAG-TT---GTGTGAC-AC-G-GAAGCGGCAGTCCTGGC

Clone#28. AC---------------------------------------CACGCCTGGC

Clone#29. ACGCCCGTTGGGCAG-TT---GTGTGAC-AC-G-GAAGCGGCAGTCCTGGC

Clone#30. ACGCCCGTTGGGCAG-TT---GTGTGAC-AC-G-GAAGCGGCAGTCCTGGC

Clone#31. ACGCCCGTTGGGCAG-TT---GTGTGAC-AC-G-GAAGCGGCAGTCCTGGC

Clone#32. ACGCCCGTTGGGCAG-TT---GTGTGAC-AC-G-GAAGC-GCAGTCCTGGC

Clone#33. ACGCCCGTTGGGCAG-TT---GTGTGAC-AC-G-GAAGCGGCAGTCCTGGC

Clone#34. ACGCCCGTTGGGCAG-TTAAG------C----G-G------CAGTCCTGGC

Clone#35. ACGCCCGTTGGGCAG-TT---GTGTGAC-AC-G-GAAGCGGCAGTCCTGGC

Clone#36. ACGCCCGTTGGGCAG-TT---GTGTGAC-ACGG-GAAGCGGCAGTCCTGGC

Clone#37. ACGCCCGTTGGGCAG-TT---GTGTGAC-AC-G-GAAGCGGCAGTCCTGGC

Clone#38. ACGCCCGTTGGGCAG-TT---GTGTGAC------G---CGGCAGTCCTGGC

Clone#39. ACGCCCGTTGGGCAG-TT---GTGTGAC-AC-GA-AA-CGGCAGTCCTGGGC

Clone#40. ACGCCCGTTG-----------------------------GGCAGTCCTGGC

Clone#41. ACGCCCGTTGGGCAG-TT---GTGTGAC-AC-G-GAAGC-GCAGTCCTGGC

Clone#42. ACGCCCGTTGGGCAG-TT---GTGTGAC-AC-G-G---CGGCAGTCCTGGC

Clone#43. ACGCCCGTTGGGCAG-TT—AC-TGTGAC-AC-G-GAAGCGGCAGTCCTGGC

Clone#44. ACGCCCGTTGGGCAG-TT---GTGTGAC-AC-G-GAAGC-GCAGTCCTGGC

Clone#45. ACGCCCGTTGGGCAG-TT---GTGTGAC-AC-G-GAAGCGGCAGTCCTGGC

<table>
<tr><td colspan="3">Total clones sequenced
45</td></tr>
<tr><td rowspan="2">Wildtype
37.8%</td><td colspan="2">Mutations
89.5%</td></tr>
<tr><td>InDels
42.2%</td><td>Scarless point (G→C) mutations
20.0%</td></tr>
</table>

Supplemental Table 7. Primers used for off-target analysis

Primer Name	Sequence(5' to 3')
B2M-offtarget1-F	TACCCAAGGGGATTTTTGGT
B2M -offtarget1-R	GATGGAAGTGAGGTGGGAAA
B2M -offtarget2-F	ACCAAGTTTTGGGCACAGAC
B2M -offtarget2-R	CCAAACCTGTTCCAAACGAT
B2M -offtarget3-F	TCGGTGGTTGGAGACCTAAC
B2M -offtarget3-R	CATATGCTGCAAAGCTGGTG
B2M -offtarget4-F	CGACAGCAGAAATGAAACCA
B2M -offtarget4-R	CTCCCAAAGTGCTGGGATTA
B2M -offtarget5-F	CCATAACCCCAGAGTCCAGA
B2M -offtarget5-R	ACCACGAGTTTTTGGAATGC
B2M -offtarget6-F	TAATGGGGTGGGGATGATTA
B2M -offtarget6-R	AAAGGGAAGAGGGCAAAAAG
B2M -offtarget7-F	CGACAAGCCAAAGAGAAAGG
B2M -offtarget7-R	GCTGGTCTTGAACTCCCAAC
B2M -offtarget8-F	TTTCAGCCTCCAGGTCTCAT
B2M -offtarget8-R	GGCACCTGTAGTCCCAGCTA
B2M -offtarget9-F	CTGCACTTTTCCCCATGTCT
B2M -offtarget9-R	CGAATTCTGGCTCTCCTGTC
B2M -offtarget10-F	AGGGGTCCTGGCTCTTCTAA
B2M -offtarget10-R	AACTGGGGCTCTATGTGTGC
AAVS1 -offtarget1-F	GTGTCCTCCTGCCAGTTAGC
AAVS1 -offtarget1-R	GTGGGACATCTCCTCAATGG
AAVS1 -offtarget2-F	CCGTACACGCTTCCTCTCTT
AAVS1 -offtarget2-R	GCATTGAGGGGGTTGAATAA
AAVS1 -offtarget3-F	AAGCCACAGCTTGAGATGGT
AAVS1 -offtarget3-R	TCAAATCTGGTCCTGGGAAG
AAVS1 -offtarget4-F	AAATCCTGAGTCCCGACCTT
AAVS1 -offtarget4-R	CAGCTGGGCATCCTGTAACT
AAVS1 -offtarget5-F	GGGAATCCCAAGGGTACATT
AAVS1 -offtarget5-R	TGTTGTGTGAGCCATCCAAT
AAVS1 -offtarget6-F	CATGCTACCATCGTCACCAC
AAVS1 -offtarget6-R	TGGGATCTCTTGGGACAGAC
AAVS1 -offtarget7-F	AGGGAGCTTTGAGGGATCAT
AAVS1 -offtarget7-R	CTGCCTTGTAGGGATTCTGG
AAVS1 -offtarget8-F	CCACCTGGGTGACAGAAGAT
AAVS1 -offtarget8-R	TCGAGGTGGTCAGGCTAAAC
AAVS1 -offtarget9-F	CCTTGGCGAGAGAGAAACTG
AAVS1 -offtarget9-R	ACTTAGAGCCGAGTGGGACA
AAVS1 -offtarget10-F	AAGCCCAGCTCTTGCTTTTT
AAVS1 -offtarget10-R	TTTTCAGGCTGGTGGAAGTT

Supplemental Table 8. Sequence analysis of predicted off-target loci of sgRNAs targeting B2M and AAVS1. No off target events were detected. PAM sequence is shown in blue, mismatches in the protospacer sequence are shaded grey. #mm: number of mismatches. Ins del: insertions or deletions.

B2M

No.	genomic location	locus details	Sequence	#mm	Ins del
	sgRNA	B2M	CTCGCGCTACTCTCTCTTTCTGG		
1	chr13:54037299-54037321:-	intergenic	CCAGCGCCCCTCTCTCTTTCTGG	4	no
2	chr9:108759543-108759565:-	Intergenic	CTAGCACTCCTCCCTCTTTCTGG	4	no
3	chr1:149186941-149186963:-	Intergenic	CCCGCGCGGATCTCTCTTTCCGG	4	no
4	chr1:43304542-43304564:+	Intergenic	CTCACTCTTCTCTCTCTTTCTGG	3	no
5	chr10:132979480-132979502:-	intron:TCERG1L	CTGGCCCCACTCTCTCTTTCTGG	3	no
6	chr2:136967279-136967301:-	Intergenic	CTCAGGCCTCTCTCTCTTTCTGG	4	no
7	chr6:136292647-136292669:-	Intergenic	CACATGCTGCTCTCTCTTTCTGG	4	no
8	chr9:98142180-98142202:-	Intergenic	CTCGGGCAATTCACTCTTTCTGG	4	no
9	chr11:80544976-80544998:-	Intergenic	CCCAAGCTACTCTCTCCTTCAGG	4	no
10	chr7:125792497-125792519:+	Intergenic	CTCATGCTGCTCCCTCTTTCTGG	4	no

AAVS1

No.	genomic location	locus details	Sequence	#mm	Ins del
	sgRNA	AAVS1	GCCAGTAGCCAGCCCCGTCCTGG		
1	chr15:101451478-101451500:-	intergenic	GCCAGAAGCCAGCCCAGTCCAGG	2	no
2	chr19:3981161-3981183:-	intron:EEF2	CCCAGCAGCCAGCCCCATCCGGG	3	no
3	chr6:155228623-155228645:-	Intergenic	TTCAGGAGACAGCCCCGTCCTGG	4	no
4	chr22:49992857-49992879:-	Intergenic	CCCTGCAGCCAGCCCCGTCCTGG	3	no
5	chr9:73825187-73825209:-	intron:TRPM3	GCAACTAGGCAGCCCCATCCGGG	4	no
6	chr3:127723709-127723731:+	Intergenic	GCCTCTACCCAGCCCCGTCCTGG	3	no
7	chr2:95673621-95673643:+	Intergenic	GACAGCCGCCAGCCCAGTCCTGG	4	no
8	chr1:38102362-38102384:+	Intergenic	GCAAGTGGCCAGCCCAGTTCAGG	4	no
9	chr1:1293446-1293468:+	intron:MXRA8	GTCAGCAGCCAGCCCAGGCCTGG	4	no
10	chr4:15907742-15907764:-	Intergenic	GCCAGAGGCCAGCCCCGCCCGGG	3	no

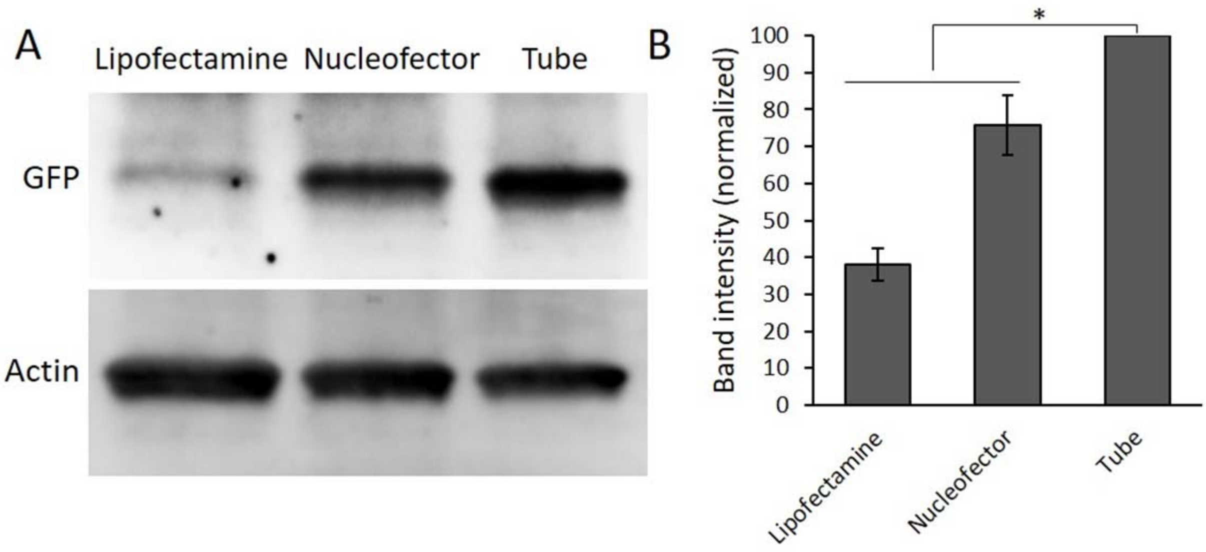

Supplemental Figure 1. Comparison of the protein expression levels after DNA transfection with different methods. HEK293 Cells were tranfected with pCMV-GFP using lipofectamine, nucleofector or tube electroporation. Cell extractions were prepared after 48 hours. Western blot (A) was used to measure the amount of GFP protein expression and quantified (B) to compare.

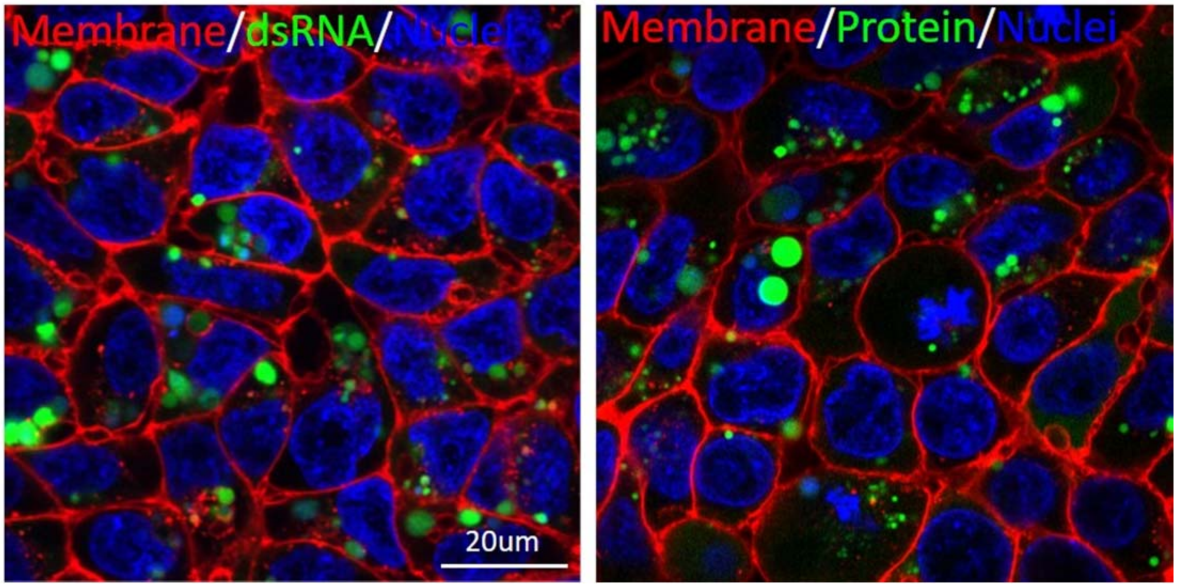

Supplemental Figure 2. Confocal microscopy images of the dsRNA and protein transfected into human iPSC. The images showed that dsRNA and IgG protein were successfully delivered inside nearly every cell.

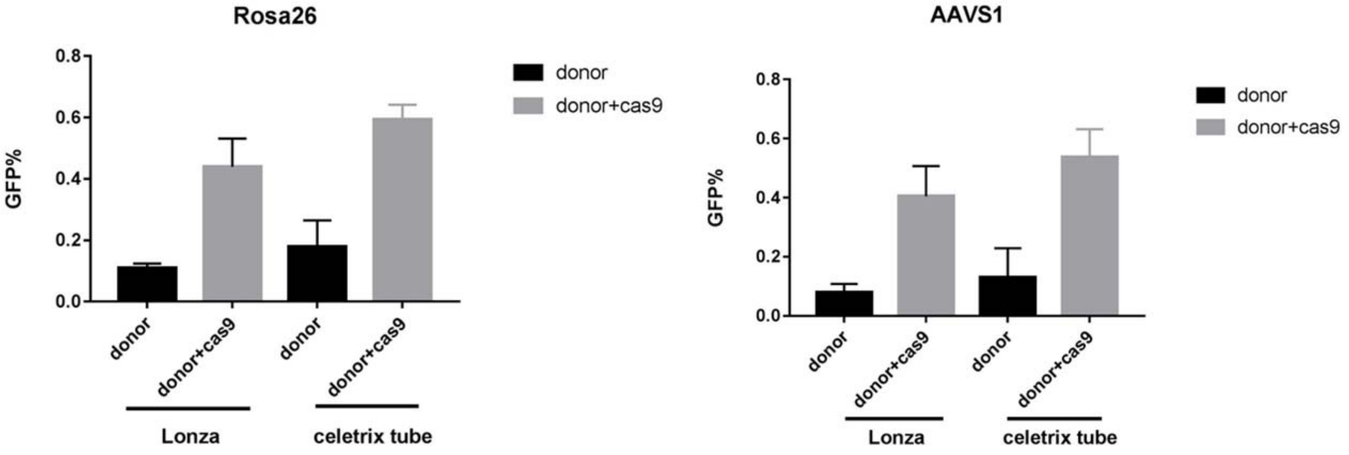

Supplemental Figure 3. Efficiencies in reporter knock-in to safe harbor loci in human iPSCs by Celetrix's tube electroporation and Lonza's Nucleofector. Left: knock-in of EGFP plasmid DNA reporter vector to Rosa26 locus. Right: Left: knock-in of EGFP plasmid DNA reporter vector to AAVS1 locus.

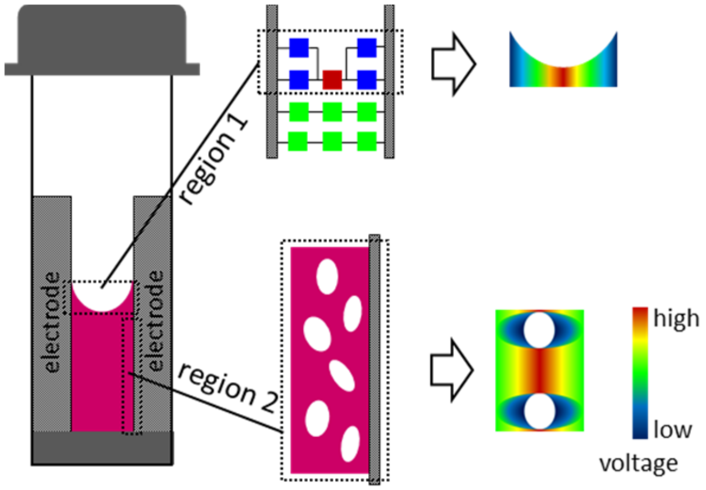

Supplemental Figure 4. Illustration of adverse physical effects associated with a conventional cuvette. The voltage distribution in a conventional cuvette is highly uneven in two regions. Region 1: the top concave meniscus. The voltage distribution can be simulated by a simplified equivalent circuit, in which identical resistors are connected into multiple series in parallel with the same end-to-end total voltage. Since the middle top resistor is missing the two side resistors (blue) are first connected in parallel and then connected in series to the center resistor (red). Therefore the voltage in the edge areas is lower than average while it is significantly higher in the middle region beneath the curve; Region 2: the electrode surface areas. The areas contain numerous electric resistant air bubbles as a result of electrochemical reactions. The areas on both sides of each bubble have lower voltage, while the electrons are forced through the spaces between bubbles creating very high voltage regions.

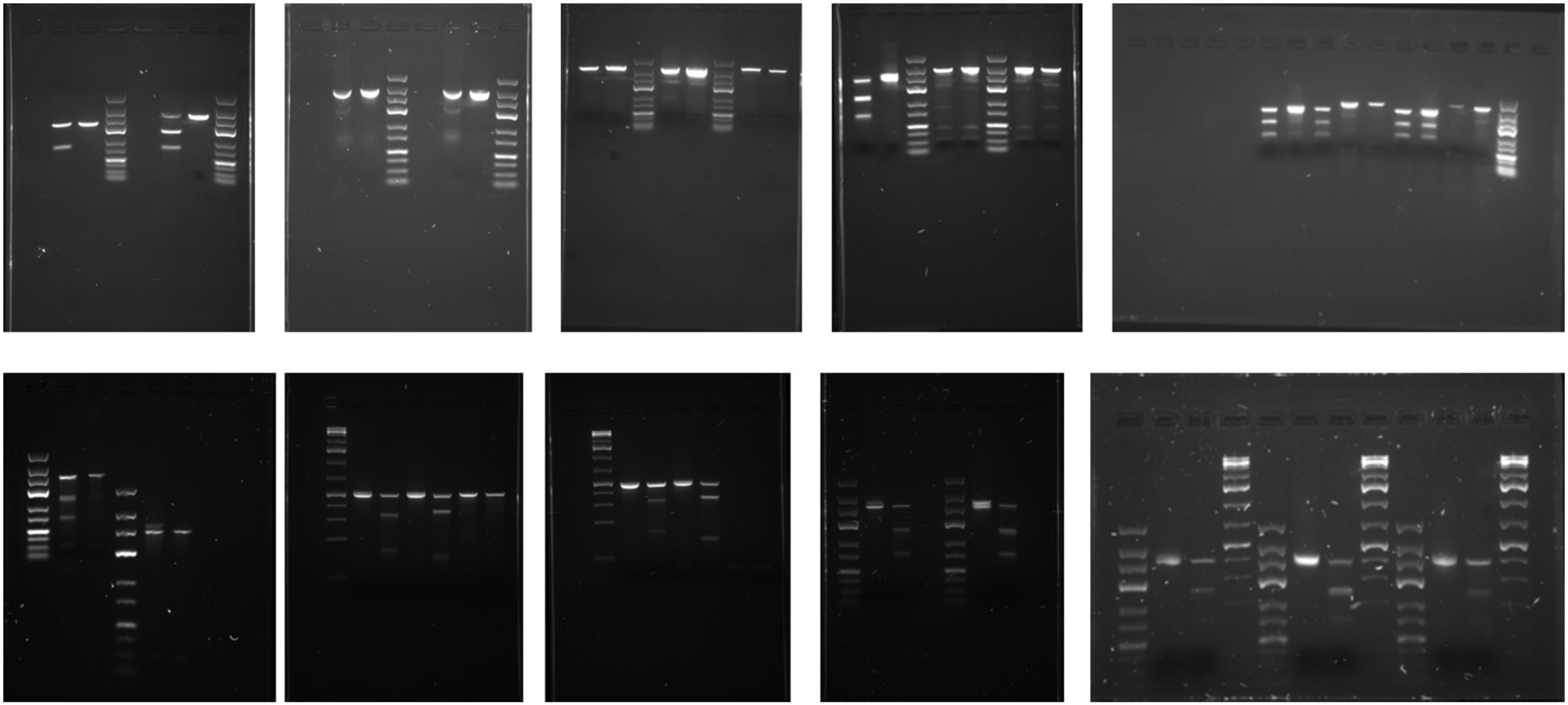

Uncropped gel pictures of Figure 4

SOLDNER F, HOCKEMEYER D, BEARD C, GAO Q, *et al.* (2009). Parkinson's disease patient-derived induced pluripotent stem cells free of viral reprogramming factors. Cell 136, 964-77.
